# Subduction-like process in Europa’s ice shell triggered by enhanced eccentricity periods

**DOI:** 10.1126/sciadv.adq8719

**Published:** 2025-06-04

**Authors:** Martin Kihoulou, Gaël Choblet, Gabriel Tobie, Klára Kalousová, Ondřej Čadek

**Affiliations:** ^1^ Nantes Université, Univ Angers, Le Mans Université, CNRS, Laboratoire de Planétologie et Géosciences, LPG UMR 6112, 44000 Nantes, France.; ^2^ Department of Geophysics, Charles University, Faculty of Mathematics and Physics, V Holešovičkách 2, Praha 8, 180 00, Czech Republic.

## Abstract

Jupiter’s icy moon Europa has a liquid water ocean beneath the ice shell and a geologically young surface, both related to extensive tidal processes. Interlaced by tectonic faults, the surface records convergent motions that induced crustal disappearance in some areas. Subduction has been advocated as an explanation; however, its driving mechanism still remains unexplained. We perform numerical simulations to test under what conditions may lateral compression initiate subduction in Europa’s ice shell. We demonstrate that subduction-like recycling can occur only for shells of thickness ≲10 kilometers, transporting the surface ice into the subsurface ocean. We further show that compression large enough to initiate subduction-like behavior in a thin shell is achieved during brief periods of enhanced eccentricity, implying increased hydrothermal activity at the seafloor. Together with the subduction-like transport of surface oxidants, such conjunction should favor chemical disequilibria in the ocean, increasing its astrobiological potential.

## Teaser

Subduction in the ice shell of Jupiter’s icy moon Europa might be triggered by variations in the moon’s orbital eccentricity.

## INTRODUCTION

Only until recently, the Earth has been considered the only body in the Solar System with active plate tectonics. When apparently analogical plate tectonics–like behavior was identified in a limited set of regions on Europa, many similarities, but also differences between the tectonics of these two bodies emerged. While on Earth, the amount of crust produced by spreading at the oceanic seafloor is balanced by convergence at subduction zones (*1*), the geological activity on Europa is dominated by extensional features with only a few areas where crustal disappearance has been detected (*2*–*6*). Moreover, divergent and convergent boundaries on Europa do not seem to be active simultaneously, and not at present day, suggesting an episodic evolution (*6*). Overall, Europa’s ice shell probably does not represent a steady state. According to thermal equilibrium–based theoretical predictions, its current thickness could typically range between 20 and 50 km, depending on whether the shell is in a conductive or convective regime (*7*–*9*). Yet, interpretations of the surface morphology suggest that the shell might have been as thin as 5 to 10 km in a recent past (*10*, *11*). Such temporal variations in Europa’s ice shell thickness are likely a consequence of changes in orbital eccentricity due to tidal interaction with neighboring Io (*12*). This indicates that Europa should alternate between periods of high heat production/thin shell and low heat production/thick shell, accompanied by global contraction and expansion, respectively, to changing volume of the hydrosphere. While previous modeling works investigated faulting during an extension phase (*13*, *14*), here, we explore the dynamics of Europa’s ice shell during a contraction phase, focusing on downward transport of near-surface ice.

## RESULTS

### Contraction of Europa’s ice shell

We model Europa’s ice shell as a rectangular domain with length of 100 km and thickness as the main model parameter, ranging from 5 to 30 km. The top boundary represents the surface of the ice shell and can evolve freely, and the bottom boundary represents the interface between the ice shell and ocean, where we assume that any topographic change is immediately compensated by melting and crystallization (*15*–*17*). On the lateral boundaries, we prescribe material influx mimicking compressive stress. Opening velocities of dilational bands have been estimated to range from 0.2 to 40mmyear^−1^ (*18*). Assuming that contraction and extension are of comparable amplitude (discussed below), we adopt a conservative value of 10mmyear^−1^.

To address both ductile and brittle deformation of the ice, we use visco-elasto-plastic rheology. Deformation in the lower, warmer part of the shell is governed by a temperature- and stress-dependent viscous creep (*19*), whereas in the cold rigid part by elasticity and brittle faulting (*20*), resulting in highly nonlinear rheology. Compared to previous studies dedicated to contraction of Europa’s shell (*21*, *22*), we account for strain weakening (*20*) and for the interaction between the ice shell and subsurface ocean (*16*). More details on the numerical model are provided in Materials and Methods and in Supplementary Text.

**Fig. 1.**
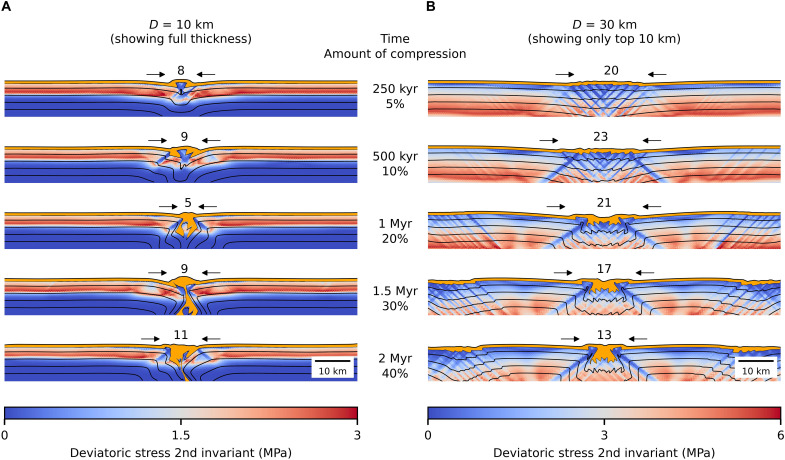
Evolution of the ice shell under compression and fate of near-surface ice. The color field represents the second invariant of the deviatoric part of Cauchy stress tensor, black lines connect material of equal initial depth (intervals of 2 km), and orange layer indicates the current position of originally near-surface ice. Black arrows delimit a feature that might be interpreted as a convergence/subsumption band; the number within indicates its width in kilometers. (**A**) Shell thickness of 10 km. (**B**) Shell thickness of 30 km.

Figure 1 compares the time evolution of a thin (10 km) and thick (30 km) conductive ice shell under compression. Shortly after the compression starts, the first faults develop (Fig. 1, top). While in the thin shell, only one pair of major faults emerges, a cluster comprising several pairs of faults forms in the thick shell. This initial distribution of faults then affects the way the near-surface ice (orange layer) proceeds downward. For the thin shell (Fig. 1A), a single pair of faults accommodates all the deformation. First, the ice slides along both sides of the fault [250 to 500 thousand years (kyr)] to later connect into a narrow descending column [1 to 1.5 million years (Myr)]. The near-surface ice then reaches the subsurface ocean after less than 30% of contraction (1.5 Myr). At the end of the simulation (2 Myr), the transport to the subsurface ocean is nearly vertical, as indicated by the opened black lines.

**Fig. 2.**
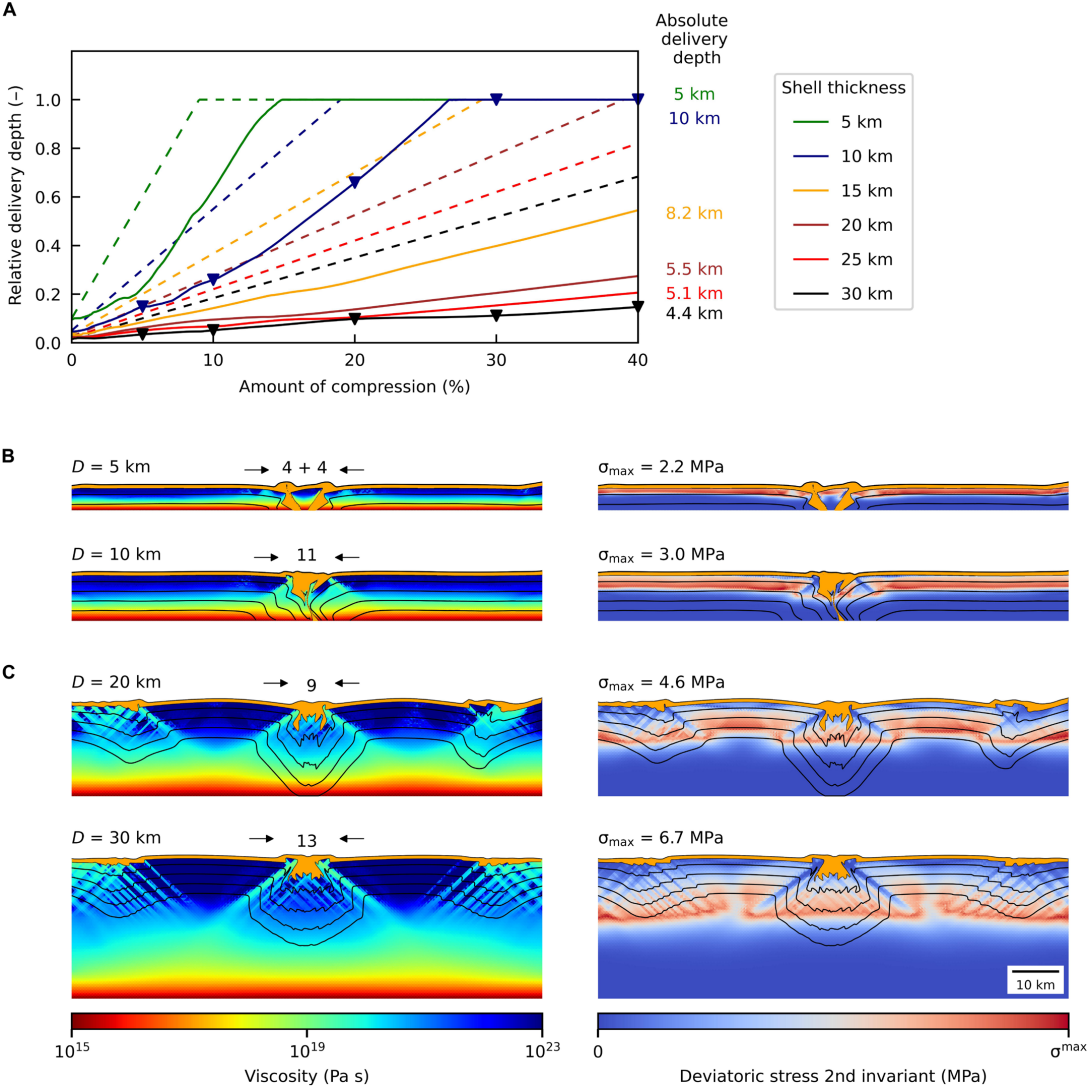
Evolution of the delivery depth. (**A**) Solid lines show the relative depth of the surface material (1 = delivery to the subsurface ocean) with respect to the amount of compression. Dashed lines show the optimal efficiency (i.e., if the descent and compressional velocity were equal). Numbers at the end of solid curves denote the absolute delivery depth for a simulation of corresponding color. Triangles from left to right correspond to snapshots in Fig. 1 from top to bottom. (**B**) Snapshots of 5- and 10-km-thick ice shells at the end of simulation (40% of compression). In these cases, the maximum downward transport velocity was close to the velocity of compression. (**C**) Snapshots of 20- and 30-km-thick ice shells at the end of simulation.

In case of the thick shell (Fig. 1B), the faults reach larger depth, as the brittle part of the shell is thicker, and are grouped in wide clusters (tens of kilometers). Deformation is accommodated by a larger number of faults, which results in less efficient sinking of near-surface ice. After 40% of compressive strain (2 Myr), the near-surface ice only reaches less than 5 km of depth (Fig. 2).

As shown in Fig. 2A, illustrating the relative depth of the deepest near-surface ice marker, the near-surface ice can be transported to depths of 5 km and more after a total compressive strain of 40% but can reach the ice-water interface only for thicknesses of ≲10 km (Fig. 2B). For shells thicker than ~10 km, the descent rate becomes substantially slower than the compressive velocity (dashed lines). This behavior can be explained by the morphology of the convergence sites (see above) and by the development of secondary faulting sites, which further decrease the descent rate (Fig. 2C). Numerical simulations with longer domains show that the secondary sites emerge usually ~40 to 60 km from the primary sites (see fig. S5). Therefore, their positions in Figs. 1B and 2C rather coincide with the corners of the 100-km-long domain. Last, for a 10-km-thick shell, we further investigated the sensitivity to the grain size, compression rate, magnitude of tidal dissipation rate, and healing of the faults. None of these parameters did substantially change the descent rate (see fig. S6).

**Fig. 3.**
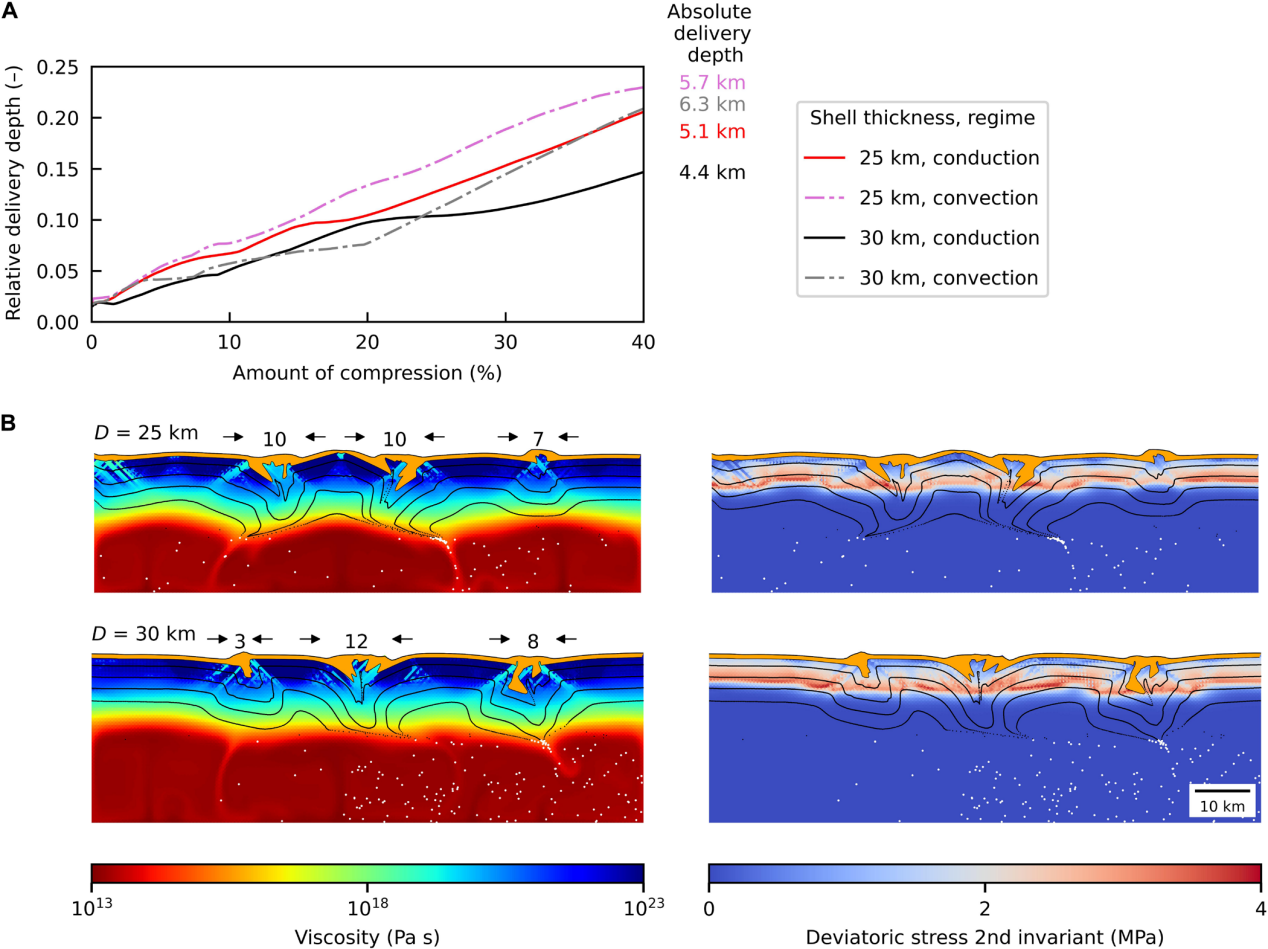
Effect of the heat transfer regime on the evolution of delivery depth. (**A**) Comparison of the relative delivery depth evolution for a convective shell (dashed lines) and conductive shell (solid lines). Numbers at the end of the curves denote the absolute delivery depth for a simulation of corresponding color. Note that the *y*-axis range differs from that of Fig. 2A. (**B**) Convective shells with thicknesses of 25 and 30 km after 40% strain. For better visibility, markers in the convective region are plotted in white.

The question of heat transfer regime within Europa’s ice shell is still a matter of debate as the parameters controlling the ice viscosity are poorly constrained. Yet, there are geological indications (e.g., chaos terrains or lenticular features) that suggest a regional solid-state convection or diapirism (*23*). To assess the effect of convection on the material descent, we performed two simulations with shell thicknesses of 25 and 30 km, assuming fully developed convection with basal viscosity equal to $10^{13}$ Pa s; see Fig. 3. Compared to a conductive shell with basal viscosity of $10^{15}$ Pa s, the near-surface ice sinks slightly faster in the convective case (Fig. 3A).

Convection effectively reduces the thickness of the cold rigid layer, which results in narrower convergence sites and larger delivery depths, despite the fact that the faults develop at multiple locations because of time-varying convective stresses. As demonstrated by white markers in Fig. 3B, once the ice from the rigid layer reaches the convective region, it is captured and dispersed by the flow and ultimately delivered in the subsurface ocean (for time sequence see fig. S7). This corresponds to the subsumption process hypothesized in (*5*). However, after 40% compression, the near-surface ice hardly reaches half the way to the top of the convective region.

### Comparison with terrestrial subduction

**Fig. 4.**
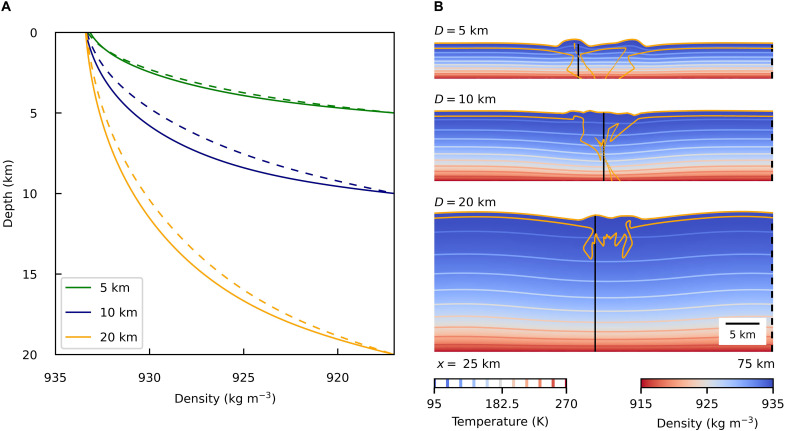
Density and temperature inside the ice shell after 40% strain. (**A**) Density as a function of depth inside (solid line) and outside (dashed line) the downwelling zone for different shell thicknesses. (**B**) Section of the density field (*x* = 25–75 km). Color contours represent temperature isolines with an interval of 17.5 K; orange lines represent the outline of near-surface ice. Solid and dashed black lines indicate the position of the respective density profiles in (A).

On Earth, the plate motions are driven by the dynamics of subduction, i.e., by a negative buoyancy resulting from the temperature difference between the cold sinking slab and warmer surrounding mantle (*1*). Because of a long diffusion time of the slab (~80 Myr, see Materials and Methods), its negative buoyancy can be preserved over a long period of time, enabling a long-term pull on the plate. However, in the environment of an ice shell, this “slab pull” is inefficient. As shown in Fig. 4, even once the subduction-like behavior is initiated, the density difference is still too small to generate negative buoyancy able to drive the sinking. Since the diffusion time of the vertically sinking portion of the ice shell is only ~100 kyr (see Materials and Methods), the descending ice rapidly balances its temperature and density with its surroundings (see Fig. 4B). Beneath the convergence site, our models predict negative density anomaly ≲ 1 kg m^−3^ (see Fig. 4A), which is less than because of the presence of salts at Europa’s surface (*24*).

As already inferred in (*24*), the subduction-like behavior on Europa must be therefore driven by a process different from the local negative buoyancy. As suggested by Figs. 1 to 4, such behavior occurs only when strong external forces maintain convergence in a thin shell during at least a few millions of years. In context of Europa, whose thermal budget is controlled mainly by tidal dissipation, such convergent forces might be provided by a contraction of the whole body as a result of enhanced orbital eccentricity (*12*, *14*).

### Compressive stress induced by thinning of the ice shell

Because of the Laplace resonance, gravitational interactions between Io, Europa, and Ganymede can lead to an increase in orbital eccentricities, potentially generating enough energy to melt Europa’s ice shell down to a few kilometers of thickness (*12*). Temporal variations of thickness have been shown to generate tensile stresses large enough to break the ice shell during thickening phases (*14*, *25*). Here, we extend the approach in (*14*) by taking into account consistently the variations in orbital eccentricity and the heat production in both the ice shell and silicate mantle. We compute the compressive stress associated with temperature change in the ice shell and volumetric changes of the hydrosphere (see Materials and Methods).

**Fig. 5.**
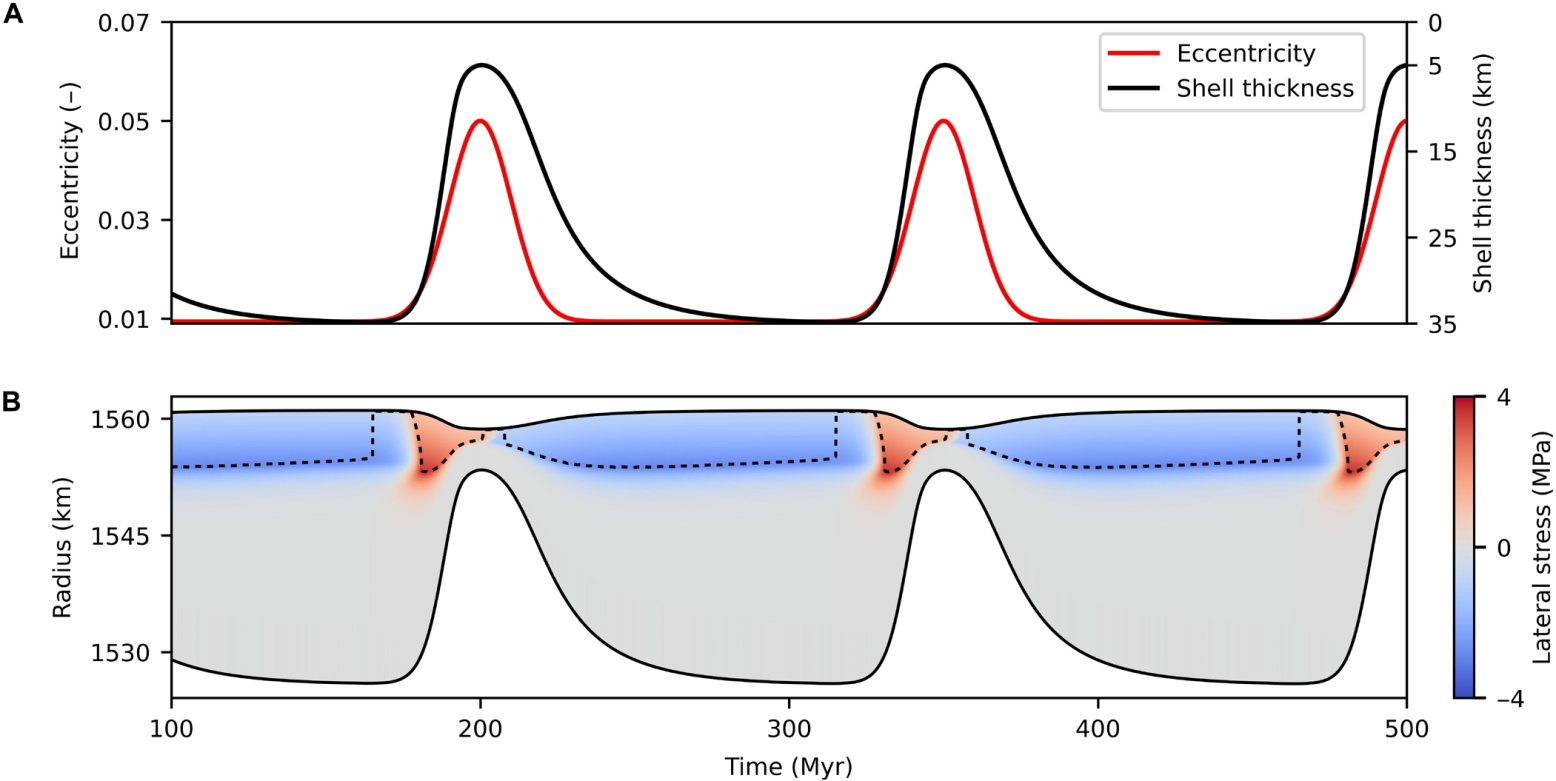
Impact of orbital eccentricity variations on stresses in the ice shell. (**A**) Prescribed orbital eccentricity (left axis, red line) and evolution of ice shell thickness (right axis, black line). (**B**) Time evolution of the lateral component of the deviatoric stress ( $\sigma_{\theta \theta }$ and equally $\sigma_{\varphi \varphi }$ ) in Europa’s ice shell. Red and blue colors represent compressive and tensile stress, respectively. Dashed contour shows the deepest part of the shell where the yield criterion $\left(\sigma_{\text{II}}=\sigma_{Y}\right)$ is met.

Figure 5 shows the evolution of lateral (compressive/tensile) stress in an initially 35-km-thick conductive ice shell in response to variations in orbital eccentricity. First, an eccentricity rising from its current value *e* = 0.0094 to a value of 0.05 yields a surge in heat produced by tidal dissipation in the silicate mantle and in the ice shell. As the ice shell gradually melts from below, compressive stress accumulates in the rigid part of the shell, up to the moment when the yield stress is reached. The deformation further continues in the elastoplastic regime (above the dashed contour in Fig. 5B) until the eccentricity starts to decrease, indicating that the shell would undergo multiple episodes of fracturing and rebuilding of the stress (not modeled here). Second, eccentricity diminution back to its current value is accompanied by the shell thickening back to its initial state and by extension that proceeds in the uppermost part of the ice shell, similar to compression. Several of such periods may successively occur. Note that the sign of the lateral stress reacts in the order of Myr (i.e., almost instantaneously) to the trend in eccentricity. For the comparison of the stress profiles between the tectonic model (Fig. 2) and the thickness evolution model (Fig. 5) during the thinning phase, see fig. S8.

According to our tectonic simulations (Fig. 2), efficient downward transport by subduction-like process will occur only once the ice shell is thin (≲10 km) and under compressive state, which coincides with periods of high eccentricity displayed in Fig. 5. Note that these conditions are already met for eccentricity of 0.03 (see fig. S4 for scenarios for different values of maximum eccentricity). As predicted by thermo-orbital models (*12*), such periods may occur every tens to hundreds of Myr and last for a few to a few tens of Myr. The apparent young age of Europa’s surface, estimated between 40 and 90 Myr (*26*), may thus represent the typical time since the last period of enhanced eccentricity. Since then, the ice shell would become progressively thicker, which appears to be consistent with the interpretation of stratigraphic sequences (*27*, *28*) and the absence of plate tectonics–like behavior at present day (*6*).

## DISCUSSION

In this study, we have conducted numerical simulations of (i) lateral compression of the ice shell with an underlying liquid water ocean and (ii) the response of the hydrosphere to variations in orbital eccentricity. Using the first model, we show that compression of the ice shell always transports near-surface ice toward the subsurface ocean, but delivers it only when the shell is sufficiently thin (≲10 km). With the second model, we demonstrate that variations in orbital eccentricity can enhance Europa’s internal heat production enough to melt the ice shell down to less than 10 km in thickness, while triggering a global compression reaching the yield stress of ice. Our results therefore link together the observations of missing surface area with thermal evolution of Europa’s interior and thus present a coherent framework for Europa’s geological history. Although the faulting and volumetric changes are treated separately in our approach, this study might set an initial step toward self-consistent modeling of Europa’s long-term global tectonic deformation.

### Driving mechanism for global contraction and expansion

Equilibrium between the creation and destruction of tectonic plates is one of the key principles of the plate tectonics and was expected to apply equally to Europa as it does to Earth. However, it is only valid under the assumption that the surface area of the body is conserved. As demonstrated in Fig. 5, during variations of ice shell thickness (here triggered by evolving orbital eccentricity), the radius and thus the surface area of the ice shell vary because of volumetric changes of the hydrosphere. Our results, along with other studies (*6*, *14*, *24*, *29*) therefore indicate that compressional and extensional structures are not a manifestation of a system of plates powered by the ice shell dynamics, but they rather emerge separately during periods of rising and decreasing eccentricity, respectively, as a result of alternating global contraction/expansion. Therefore, convergent features might have been much more common during periods of rising eccentricity/thinning the ice shell.

### The role of compression in Europa’s geology

Several geological clues, such as the predominance of ridged plains in the early period of Europa’s surface (*30*), or tidal walking on strike-slip faults, which requires a very thin shell (≤5 km) (*11*), attest to a state of high heat production/thin shell in Europa’s past. However, unlike divergent processes, the convergent processes do not generate structures that could be easily distinguishable from the surrounding terrain and are therefore much more difficult to identify on Europa’s surface. This limited tectonic record is furthermore obscured by posterior resurfacing and complex motions and rotations of the fractured surface. So far, the most established method of identification relies on the reconstruction of the surface motions based on discontinuous preexisting features (*2*–*6*, *30*). Convergent areas, nevertheless, are also often accompanied by compressional/subsumption bands (*30*, *31*), which our numerical model equally predicts (see Figs. 1 to 3). Although compression does not always imply convergence, these bands might provide more accessible evidence. High-resolution surface images and topographic data to be collected by Europa Clipper and JUICE spacecrafts may therefore reveal more compressive features from the earlier periods.

On Europa, the observed width of the most prominent subsumption bands in the Northern Falga region reaches up to 30 km (*5*, *31*), which might correspond to the initial phase of compression of a thick shell (see Fig. 1B). According to the reconstructed sequence, these wide subsumption bands are adjacent to the lost surface areas that were the first ones to be recycled (*6*). This observation supports the scenario presented in Fig. 5, suggesting that the compression of the ice shell starts during a thick shell period when the eccentricity is rising from low values. Furthermore, the loss of ~90-km-wide surface area along the Southern subsumption band indicates that the subduction-like process remained (episodically) active as the ice shell was gradually thinning. Apart from this unique event, the reconstructions also revealed a number of narrow (~km) areas of missing surface (*6*). Sequences of motions in these areas show that convergent, strike-slip and divergent motions can often alternate, indicating that a full three-dimensional approach may be necessary to properly simulate regional stress accumulation due to global contraction and associated complex plate motions.

In addition to constitute an essential test of the hypothesis proposed here, the identification of subsumption bands would provide a means to quantify shell thickness; our model shows that the width of subsumption bands reflects the span of the compressional faults, which in turn depends on the shell thickness. Thin shells, i.e., the only way for near-surface ice to penetrate deep into the ocean, imply narrow subsumption bands. Apart from the faulting, our thick-shell models (Fig. 3B) also show flexure arcs in the region between the faults with the amplitude of the order of hundreds of meters, similar to the prediction in (*21*, *22*). Note that our tectonic model does not include compressibility, which might affect morphology and distribution of the faults (*32*). However, supported only by the elastic stress, these features should emerge only during the compression phase. Their presence or absence might therefore serve as an indication of a present-day stress tendency in Europa’s ice shell.

### Geochemical implications for Europa’s subsurface ocean

On the basis of the scenario presented in Fig. 5, the shrinkage of the outer shell radius during the contraction phase should result in loss of ~90,000 km^2^ of surface area [missing area of ~16,000 to 20,000 km^2^ was reported in the Northern Falga region (*5*, *6*)]. We demonstrated that if the convergence occurs during the thin shell period (≲10 km), a portion of the near-surface ice might be transported to the subsurface ocean in less than 2 Myr. Moreover, if the convergent faults perform strike-slip motions at the timescale of diurnal tides and generate melt by frictional heating, the denser melt promotes a Rayleigh-Taylor instability and might further reduce the delivery time (*33*). Assuming a global accumulation of dioxygen (O_2_) in the icy regolith by radiolytic processes at rate of 6 to 18 $\text{kg}\;s^{-1}$ (*34*) over a typical timescale of 100 Myr, the subduction-like process would transport $0.6\;\text{to}\;1.4\times 10^{14}$ kg of O_2_ to the subsurface ocean during a period of 2 Myr. This would correspond to $0.9\;\text{to}\;2.6\times 10^{9}$ $\text{mol}\;O_{2}$ year^−1^ delivered to the ocean, comparable with the estimate in (*35*), however, without the need for complete resurfacing. Moreover, the likely presence of other oxidants ( $H_{2}O_{2},\;CO_{2},\;SO_{2},\text{and}\;SO_{4}^{2-}$ ) trapped in the regolith (*35*, *36*) will further increase the oxidants flux. Such delivery of oxidants during periods of enhanced eccentricity would be accompanied by an increase of mantle dissipation by about one order of magnitude and more, and therefore by a strong increase of seafloor hydrothermal activity (*37*, *38*). Efficient transport of surface oxidants in conjunction with enhanced hydrothermal flux might then constitute the most favorable context for ignition of complex chemistry in Europa’s subsurface ocean (*39*). Our prediction presents a lower bound of the range $10^{9}\;\text{to}\;10^{11}$ $\text{mol}\;O_{2}\;$year^−1^ proposed on the basis of simpler models for surface recycling (*35*, *36*). Even if moderate, the recycling of surface oxygen during peak activity of the subduction-like process described here may temporarily affect the oceanic chemical equilibrium (*35*). The lifespan of such O_2_-enriched periods will then depend on the frequency of eccentricity variations as well as on the rate of oxygen consumption by reductants in the ocean (*35*). Our findings will therefore motivate future works to investigate the astrobiological implications of intermittent injection of oxidants into the subsurface ocean.

## MATERIALS AND METHODS

### Tectonic model of lateral compression

To simulate thermomechanical evolution of the ice shell, we solve mass, momentum, and energy balance using constitutive equation for Maxwell viscoelastic medium in the following form

∇·v=0 (1)

$$\begin{array}{c} -\nabla p+\nabla \cdot \sigma +\rho g=0 \end{array}$$ (2)

$$\begin{array}{c} \sigma -\eta \left(\nabla v+\nabla^{T}v\right)=-\frac{\eta }{\mu }\overset{\nabla }{\sigma } \end{array}$$ (3)

ρcp∂T∂t=∇·(k∇T)−ρcp(v·∇T)+Htidal (4)

where v is the velocity, p is the pressure, σ is the deviatoric part of Cauchy stress tensor, $\overset{\nabla }{\sigma }$ its upper convective time derivative, $\nabla^{T}$ denotes the transposed gradient, ρ is the density, g is the gravitational acceleration, $c_{p}$ is the specific heat, T is the temperature, t is time, k is the thermal conductivity, $H_{\text{tidal}}$ is the tidal dissipation rate, η is the viscosity, and μ is the shear modulus.

For the momentum balance (Eq. 2), we prescribe free surface boundary condition on the top boundary ( τ·n=0 , where τ is the Cauchy stress tensor and n denotes a unit normal vector to the boundary), the hydrostatic pressure at the bottom boundary ( τ·n=−Dρgn , where D is the ice shell thickness), and convergent horizontal component of the velocity $v_{x}$ on the vertical boundaries. For the energy balance (Eq. 4), we prescribe a fixed temperature at the top and bottom boundaries and zero heat flux through the vertical boundaries. As the initial condition, we prescribe a conductive profile with a small (2 K) cosine-shaped variation, which facilitates focusing the initial faulting in the middle of the domain. The models presented in Fig. 3 and fig. S7 were started from a convective temperature field obtained by solving Eqs. 1 to 4 with viscous rheology only. Complete overview of model parameters is provided in table S1. The model is implemented in the open-source finite-element platform FEniCS (*40*, *41*) and is built upon a model of thermal convection developed by the main author (*16*), complemented with visco-elasto-plastic rheology. For verification of the code, see Supplementary Text and figs. S1 and S2.

### Visco-elasto-plastic rheology

To capture both the ductile viscous flow and brittle failure of the ice, we use visco-elasto-plastic rheology. The ductile viscosity combines four deformation mechanisms (diffusion creep, dislocation creep, basal slip, and grain boundary sliding) that can be combined in the following way (*19*)

1ηduct=1ηdiff+1ηdisl+1ηGBS+ηBS+1ηmax (5)

where $\eta_{\text{max}}$ limits the upper value of viscosity for low temperatures (upper part of the ice shell). The viscosity from the diffusion creep can be expressed as in (*19*)

$$\begin{array}{c} \eta_{\text{diff}}=\frac{\mathrm{RT}d^{2}}{3/2\cdot 84V_{m}}{\left[D_{0,v}\text{exp}\left(-\frac{Q_{v}}{\mathrm{RT}}\right)+\frac{\pi \delta }{d}D_{0,b}\text{exp}\left(-\frac{Q_{b}}{\mathrm{RT}}\right)\right]}^{-1} \end{array}$$ (6)

where R is the universal gas constant, d is the grain size, $D_{0,v}$ and $D_{0,b}$ along with $Q_{v}$ and $Q_{b}$ are the preexponential factors and activation energies for volume and boundary diffusion (see values in table S2), respectively, and $V_{m}=1.97\times 10^{-5}\;m^{3}$ and $\delta =9.04\times 10^{-10}\;m$ are the molar volume and the grain boundary width, respectively. The viscosity from the stress-dependent mechanisms (dislocation creep, basal slip, and grain boundary sliding) has the form (*42*)

η=13(n+1)/2dpAσIIn−1exp(QRT) (7)

where $\sigma_{\text{II}}$ is the second invariant of the deviatoric stress tensor. Values of the prefactor A , the activation energy Q , the grain size exponent p , and the stress exponent n depend on the specific mechanism (see table S2) (*19*).

When the stress exceeds the yield stress $\sigma_{Y}$ , the ice starts to deform by plastic deformation. Here, we adopt the Drucker-Prager criterion

$$\begin{array}{c} \sigma_{Y}=\left(p+p_{\text{global}}\right)\text{sin}\left(\phi \right)+C\cos\left(\phi \right) \end{array}$$ (8)

where p is the pressure obtained by solving Eqs. 1 to 3 and $p_{\text{global}}$ is the non-hydrostatic pressure induced by contraction of the spherical shell obtained by a priori solving of the thickness evolution model (see Eqs. 14 to 17 below). Last, ϕ is the angle of internal friction and C is the cohesion. During the plastic deformation, the material accumulates plastic strain $\varepsilon_{p}$ but can be healed by microscopic processes with typical timescale τ (*20*)

∂εp∂t=ε˙II    if σII≥σY0       if σII<σY−εpτ (9)

where ${\dot{\varepsilon }}_{\mathrm{II}}=\sqrt{\left(\dot{\varepsilon }:\;\dot{\varepsilon }\right)/2}$ is the second invariant of the strain rate tensor. Since the value of healing timescale τ is not well established, healing is not included in the nominal simulations (see fig. S6 for a simulation that includes healing). Materials such as ice or rock become increasingly weaker with the plastic damage (strain weakening), focusing the deformation into narrow faults. This behavior is usually described as

$$\begin{array}{c} C=\left\{ \begin{array}{c} C_{0}+\left(C_{\infty }-C_{0}\right)\frac{\varepsilon_{p}}{\varepsilon_{\infty }}\;\text{if}\;\varepsilon_{p}<\varepsilon_{\infty }\\ C_{\infty }\;\;\;\;\;\;\;\;\;\;\;\;\;\;\;\;\;\;\;\;\;\;\;\;\;\;\;\;\;\text{if}\;\varepsilon_{p}\geq \varepsilon_{\infty } \end{array}\right. \end{array}$$ (10)

where $\varepsilon_{\infty }$ is the value of plastic strain above which the ice is considered to be fully damaged, and $C_{0}$ and $C_{\infty }$ denote cohesion of undamaged and fully damaged ice, respectively (*43*). The viscosity in Eq. 3 is then given by $\eta =\text{min}\left(\eta_{\text{duct}},\sigma_{Y}/2{\dot{\varepsilon }}_{\text{II}}^{\text{vis}}\right)$ , where ${\dot{\varepsilon }}_{\text{II}}^{\text{vis}}$ is the viscous part of the strain rate invariant. Last, the elasticity enters the problem through the right-hand side of Eq. 3. Note that when using free surface boundary condition, elasticity should not be neglected; otherwise, the lithospheric stresses might be largely overestimated (*44*), which might directly affect plastic deformation.

### Tidal dissipation

For the tidal dissipation rate in Eq. 4, we use the formula

Htidal=2Hmaxη/ηmax+ηmax/η (11)

where $H_{\text{max}}$ is the maximum tidal dissipation rate that occurs at viscosity $\eta_{\text{max}}=\mu /\omega$ , where μ is the shear modulus and ω is Europa’s orbital frequency (*8*). For conductive models in this study (basal viscosity $10^{15}$ Pa s), the maximum tidal heating is observed at the bottom of the shell, whereas for convective models (basal viscosity $10^{13}$ Pa s), the maximum occurs above convective cells.

### Temperature-dependent material properties

In the momentum equation (Eq. 2), we assume temperature-dependent density given by $\rho \left(T\right)=\rho_{0}V\left(T_{\text{ref}}\right)/V\left(T\right)$ (*45*), where $\rho_{0}=917\;\text{kg}\;m^{-3}$ is the density at reference temperature $T_{\mathrm{ref}}=273\;K$ and V(T) is the unit-cell volume (*46*). In the heat transfer equation (Eq. 4), we use thermal conductivity given by $k\left(T\right)=k_{1}/T$ , where $k_{1}=567\;W\;m^{-1}$ (*47*) and specific heat $c_{p}\left(T\right)=c_{1}+c_{2}T$ , where $c_{1}=185\;J\;K^{-1}\;kg^{-1}$ and $c_{2}=7.037\;J\;K^{-2}\;kg^{-1}$ (*48*).

### Free surface evolution

The evolution of the top boundary is governed by the following equation

∂hs∂t=vz−∂hs∂xvx (12)

where $h_{s}$ is the surface topography and $v_{x}$ and $v_{z}$ are the horizontal and vertical components of the velocity, respectively. Evolution of the phase interface in the body’s interior presents a challenging modeling problem because of a complex interaction between the topography of the phase interface and the flow in the solid and liquid phase (*49*, *50*). The topography of the ice-ocean interface can be created by dynamic processes inside the ice shell and melting/crystallization at the interface and destructed by viscous relaxation and topography-associated water flow (*15*, *16*). Recent model of the flow in a subsurface ocean shows rather weak topography-associated flow at large wavelengths (≈1000 km) in latitudinal direction and predict that any ice-water interface topography on smaller spatial scales (≲100 km) should be rapidly erased (*17*). By assuming a fixed flat boundary, we therefore consider the ice-water interface permeable for mass and energy transfer, which corresponds to instantaneous balancing between dynamic topography building and destruction by lateral viscous flow and oceanic water flow.

### Diffusion time of a sinking slab

Cold rigid slab sinking from the surface into the warm interior of a body experiences negative thermal buoyancy, which ceases in time as the slab warms up by its surroundings. The timescale of warming can be estimated by the characteristic time of conductive heat transfer $\tau_{D}$ given by

τD=b2κ (13)

where b is the characteristic thickness of the slab and κ its thermal diffusivity. Since the ice shell is compositionally uniform in our models, we define b as the width of the column moving vertically downward at the level of cold elastic part. Using b=50km (*1*) and $\kappa =10^{-6}\;m^{2}\;s^{-1}$ (*51*) for a terrestrial slab, and b=4km and $\kappa \left(T_{s}\right)\approx 7\times 10^{-6}\;m^{2}\;s^{-1}$ for sinking ice (see Fig. 2 case D=5km and the thermal properties above), we estimate the characteristic time to ≈80 Myr and ≈100 kyr, respectively.

### Thickness evolution model

To evaluate the global stresses in the ice shell that changes its thickness because of varying eccentricity, we solve the following set of equations

∇·u=−pK+3αlΔT (14)

$$\begin{array}{c} \nabla \cdot \tau =0 \end{array}$$ (15)

$$\begin{array}{c} \sigma -\mu \left[\nabla u+\nabla^{T}u-\frac{2}{3}\left(\nabla \cdot u\right)\right]=-\int_{0}^{t}\frac{\mu }{\eta }\sigma \;dt \end{array}$$ (16)

ρcp∂T∂t=∇·(k∇T)+Htidale (17)

Here, u denotes the displacement, p is the pressure, K is the bulk modulus, $\alpha_{l}$ is the linear thermal expansivity coefficient, ΔT is the temperature departure from initial condition, τ is the Cauchy stress tensor, σ its deviatoric part, μ is the shear modulus, η is the viscosity, and $H_{\text{tidal}}^{e}$ is the eccentricity-dependent tidal heating term. We discretize Eqs. 14 to 17 using spherical harmonic functions (*52*) and finite differences in lateral and radial direction, respectively, and, assuming spherical symmetry of the problem, we solve only for degree 0. We compute the bulk modulus from the shear modulus μ and Poisson’s ratio ν as $K=2\mu \left(1+\nu \right)/\left[3\left(1-2\nu \right)\right]$ . For the momentum equation (Eq. 15), we prescribe free surface boundary condition ( $\tau \cdot e_{r}=0$ ) on the top boundary and the ocean overpressure ($\tau \cdot e_{r}=e_{r}P_{\text{ocean}}$ ) on the bottom boundary, where $e_{r}$ is a unit radial vector. The viscosity is computed in the same way as in the tectonic model; however, (i) we do not impose viscosity limiter $\eta_{\text{max}}$ and (ii) strain weakening is not included since the model assumes spherical symmetry and cannot thus capture the emergence of tectonic faults. Following (*25*), we determine the ocean overpressure iteratively, searching for such $P_{\text{ocean}}$ that solution of Eqs. 14 to 16 satisfies the condition for the surface displacement $u_{r}\left(r_{s}\right)$

ur(rs)=ΔDΔρρwf1+fΔρ/ρw,f=1−2Dini/rs (18)

where $D_{\text{ini}}$ is the initial shell thickness, ΔD is the amount of shell thickness change, Δρ is the density difference between the ice shell and water ocean, and $\rho_{w}$ is the density of water. The evolution of ice shell thickness is given by the discontinuity of the heat flux at the ice-water interface

Δr=qi−qoρLΔt (19)

where $q_{i}=\left(-k\nabla T\right)\cdot e_{r}$ is the radial heat flux in the ice shell at the ice-water interface, $q_{o}$ is the radial heat flux coming from the interior (radioactive decay and tidal dissipation in the silicate mantle, see below), L is the latent heat of water ice, and Δt is the time step. Comparison of the problem of thickening ice shell (*25*) with the solution in (*14*) led to a satisfactory agreement (see Supplementary Text and fig. S3).

To trigger changes in the thickness of the ice shell, we prescribe variations in Europa’s orbital eccentricity

$$\begin{array}{c} e\left(t\prime \right)=e_{0}+\left(e_{\text{max}}-e_{0}\right)\sum_{n=1}^{4}\text{exp}\left\{-\frac{{\left[t^{\prime }-\left(50+150n\right)\right]}^{2}}{200}\right\} \end{array}$$ (20)

where t′ is time in Myr, creating four Gaussian pulses with minimum and maximum values of $e_{0}$ and $e_{\text{max}}$ , respectively, with the center of the first pulse at 50 Myr and repeating every 150 Myr. The tidal dissipation rate in the ice shell depends on the eccentricity as

Htidale=Htidal(ee0)2 (21)

where the present-day volumetric dissipation rate $H_{\text{tidal}}$ is given by Eq. 11 and $e_{0}$ is the present-day eccentricity (*8*). The heat generated in the mantle is efficiently transported through the ocean to the bottom of the ice shell, resulting in a heat flux

qo=14πrb2[Prad+Ptidal(ee0)2] (22)

where $r_{b}$ is the radius of ice-water interface and $P_{\text{rad}}$ with $P_{\text{tidal}}$ represent the total present-day radiogenic and tidal heat production in the mantle, respectively (*38*). The tidal dissipation in the ocean is most likely negligible because of its large thickness (*53*).

Starting from an initial state at equilibrium with present-day eccentricity, we prescribe temporal variations in orbital eccentricity (Eq. 20) and within each time step, we adjust the heat sources (Eqs. 21 and 22), update the position of the bottom boundary (Eq. 19), solve the governing equations (Eqs. 14 to 17), and move the computational grid by the radial part of the displacement. The model parameters are provided in table S4.

## Supplementary Materials

### This PDF file includes:

Supplementary Text Figs. S1 to S10 Tables S1 to S4 References
