# Supplementary material for: Subduction-like process in Europa’s ice shell triggered by enhanced eccentricity periods
Source: Sci Adv. 2025 Jun 4;11(23):eadq8719. doi: 10.1126/sciadv.adq8719 (PMC12136034; doi:10.1126/sciadv.adq8719)
Supplement: Supplementary file 1 — Supplementary Text Figs. S1 to S10 Tables S1 to S4 References [file sciadv.adq8719_sm.pdf]

Science Advances
AAAS

Supplementary Materials for

Subduction-like process in Europa's ice shell triggered by enhanced eccentricity periods

Martin Kihoulou *et al.*

Corresponding author: Martin Kihoulou, martin.kihoulou@mff.cuni.cz

Sci. Adv. **11**, eadq8719 (2025)
DOI: 10.1126/sciadv.adq8719

This PDF file includes:

Supplementary Text
Figs. S1 to S10
Tables S1 to S4
References

Supplementary Text

<u>Comparison of our numerical model of lateral compression and extension to other models</u>

The numerical code presented in this study was developed using the open-source finite-element platform FEniCS (40, 41) with an Arbitrary Lagrangian-Eulerian method for evolution of the free surface (54), and Lagrangian particles (markers) for advection of elastic and plastic properties. In order to validate the implementation of the visco-elasto-plastic rheology, we performed comparative simulations using the ASPECT mantle convection code that uses field method for advection (55–57) and the SiStER geodynamic modeling code that uses finite differences for spatial discretization and sticky air method for free surface evolution (58). For the sake of comparison, we employed a simplified setting assuming constant temperature properties, zero tidal heating, viscosity given by $\eta(T) = 0.5A^{-1}\exp(Q/RT)$ and a constant time step of 0.5 kyr. The time step value, smaller than both conductive and convective time steps, ensures that the faults form with a width of one cell/element. Larger time steps result in slower plastic strain accumulation, hence more diffuse faults. While Tables S1 and S2 summarize the parameters for the model in the main text, Table S3 lists the parameters that were modified for the comparative simulations performed here.

We performed simulations of extension (Fig. S1) and compression (Fig. S2) of the ice shell. All three codes predict a major pair of faults that emerges in the elastic part of the shell. Subsequently, a wedge-shaped segment delimited by these faults further subsides in case of extension or rises in case of compression. The major faults are always accompanied by secondary ones, their position and extent slightly differ between the models. In the vicinity of tectonic faults, topographic changes between two neighboring elements can reach a significant fraction of their height, which can lead to distortion of the computational mesh after several time steps. Therefore, a stabilization of the topography is required. In ASPECT, the "Hillslope transport coefficient", intended to mimic erosional/depositional processes, can be prescribed to smooth out the topography. This, however, results in a smoother topography profile than in our simulation (see Figs. S1B and S2B). In our code, we address this issue by detecting oscillations of the topography and solving their diffusion only in the affected parts until the oscillations are smoothed sufficiently. This procedure enables us to reach steeper topography changes without smoothing the rest of the topography (see Figs. S1A and S2A). This might affect the distribution of secondary faults within the shell and explain why the development of asymmetric secondary faults occurs slightly earlier in the FEniCS simulation than it does in the ASPECT simulation.

The SiStER code uses a fixed finite-difference grid and therefore does not suffer from oscillations, as the surface of the shell is represented by the interface between the ice and the sticky air layer. Yet, it appears that the interior of the domain systematically cools down during the simulations

(see Figs. S1C and S2C). We observe that this effect becomes stronger with shortening the time step, however, for the value used here, it does not seem to affect the brittle faulting substantially. Moreover, different distribution of secondary faults in the compression case might be related to the tilted surface close to the left and right boundary, most likely a consequence of the treatment of the influx of markers for the ice and the sticky air layer. Overall, we show that all three codes produce very similar qualities of the brittle faults and we believe that this comparison proves correct implementation of faulting in our numerical model.

Validation of the numerical model of melting/freezing shell in spherical geometry.

The numerical model for computation of stresses in a freezing/melting spherical ice shell was tested on a problem of initially thin, thickening shell (25). Figure S3 shows the profiles of tangential stress resulting from the temperature change effect (Fig. S3A) and volume change effect (Fig. S3B), together with the temperature profile in the elastic part of the shell ($T < 180$ K). Our solution is very similar to that of (25), however, we obtain slightly higher stresses in the elastic lithosphere. This might be related to a lower grid resolution and to use of analytical Stefan solution for solidification of the ocean by (25). This same observation was reported by the previous study (see Figs. S5a and S6a in (14)), with the results of which we obtained a perfect match.

Figure S4 extends Fig. 5 from the main text by showing evolution of the stress in the ice shell for different values of maximum orbital eccentricity. As shown in Fig. S4, due to the thermal inertia of the ice shell, there is a lag between the eccentricity peak and the minimum thickness/maximum stress in the shell. While the previous work prescribes the time-varying heat flux at the base of the shell assuming sinusoidal variations (14), we calculate it from the radiogenic decay, tidal dissipation in the silicate mantle and from the imposed eccentricity change (38). Moreover, we compute the tidal dissipation in the ice shell from the conductive temperature profile, which further reduces the heat flux at the base of the ice shell, and therefore yields larger changes in thickness. For eccentricity higher than 0.04, the efficiency of shell thinning drops, as increasingly higher heat flux is required to melt a given amount of ice. Since our model predicts that the yield stress is reached very soon (already at $e = 0.013$) and persists until the eccentricity changes its trend again, the stress in the first several kilometers may be released by brittle failure (14) and therefore repetitive breaking of the shell and building of the stress is expected.

Effect of the domain width on the location of secondary faulting sites.

In order to maintain the same background strain rate for a fixed compression velocity, all the models presented in the main text were computed with equal domain width of 100 km. The thick-shell models developed secondary faulting sites close to the top corners of the domain aside from the primary ones in the middle, which might be attributed to the effect of the vertical boundaries (see Fig. 2C).

To assess the effect of the vertical boundaries on the location of secondary faulting sites, we performed simulations with shorter and longer domains than in the main text (100 km), namely (a) 60×20 km, (b) 60×30 km, (c) 140×20 km, (d) 180×20 km, (e) 180×30 km and (f) 210×30 km. Figure S5 shows the distance between primary and secondary faulting sites for the cases listed above. In simulation (a), a single secondary fault developed from the right corner of the domain. In the case of simulation (b), no secondary faulting site emerged. Simulations (c) and (d) both developed two primary sites close to the center, and two secondary sites. In simulation (c), one of them is located in the left corner, while the other one ~20 km from the right corner. For simulation (d), both secondary sites lie ~40 km away from the corners and ~40 km away from the primary sites. Similar situation applies to cases (e) and (f), where the secondary faults are always at least 30 km distant from the corners and 50 – 60 km distant from the primary sites.

This shows that if the domain is sufficiently long, the secondary faulting sites are not affected by the boundary condition, but they indeed emerge within 40 – 60 km from the primary sites. Therefore, in case of simulations shown in Fig. 2C, their location rather coincides with the corners of the 100-km-long domain.

Effect of secondary parameters on the evolution of relative delivery depth.

Figure S6 shows the sensitivity of the tectonic model to the secondary parameters (grain size, compression rate, magnitude of tidal dissipation rate and healing of the faults) compared to the reference case (D = 10 km) presented in the main text (Fig. 2).

Grain size

The model with grain size 0.1 mm shows slightly faster delivery (~10 kyr) than the reference case. Due to smaller grain size, the brittle part of the shell is thinner, and therefore it takes less time to reach the ductile part of the shell, where the transport is nearly vertical (hence the most efficient). Note that a secondary pair of faults emerged at ~700 kyr and decreased the speed of descent at the primary fault (see Fig. S6A, green line). In contrast to smaller grain size, the use of larger grain size 10 mm results in thicker brittle part of the shell. It takes therefore longer time to reach the

ductile part and the delivery occurs ∼60 kyr later with respect to the reference model (see Fig. S6A, violet line).

Healing of the faults

The model with healing of the faults is the only one notably faster than the reference case, delivering the near-surface ice by 200 kyr earlier (see Fig. S6A, orange line). The typical central pair of faults emerged, however, because of the healing, the convergence at the right fault was temporarily inhibited. All the convergence was therefore accommodated only by the left fault (the sunken near-surface ice is much more asymmetric than other cases, see Fig. S6B), which implies faster delivery.

Compression speed 10 km/Myr

The model with ten times smaller compression rate was the only one that did not deliver near-surface ice before reaching 40% compression (see Fig. S6A, brown line). During the simulation (at ca 15% of the compression), two additional faulting sites develop, which gradually slows down the delivery rate, since the convergence occurs at all three faulting sites at the same time. Note that even though a slower compression velocity (hence smaller strain rate) is prescribed, the stress in the shell is still large enough to fracture the ice. However, the peak deviatoric stress is smaller by ca 400 kPa compared to the reference case, since the nonhydrostatic pressure due to slower convergence rate is smaller.

Increased tidal heating

Finally, the model with increased tidal heating does not visibly change the delivery rate (see Fig. S6A, gray line). Since the peak tidal heating occurs at the bottom of the shell and rapidly decreases towards the surface, its effect on the conductive temperature profile, and therefore on the simulation, is rather low.

<u>Consistency of the stress between the tectonic model and the thickness evolution models.</u>

To support the consistency of the tectonic and the thickness evolution models, we compare the stress-depth profiles for selected values of ice shell thickness (see Fig. S8). In both models, the stress starts at 1 – 2 MPa at the surface and increases until it reaches its maximum at the bottom of the elasto-plastic layer. For the tectonic model, the maximum occurs between 2 and 6 MPa, for the thickness evolution model between 1 and 4 MPa, depending on the shell thickness. Between the two models, the maximum occurs at a very similar depth. Deeper in the ductile part of the shell, the stress rapidly decreases down to < kPa.

Figures S9 and S10 show selected properties (pressure, lateral component of the deviatoric stress, second invariant of the deviatoric stress and the second invariant of the strain rate) in the tectonic and thickness evolution models, respectively. For the tectonic model, we show the full pressure, whereas for the thickness evolution model only the non-hydrostatic part. Although the lateral stress in the tectonic model is mostly compressive, the character changes into tensile in the vicinity of the faulting site. This is most likely due to bending of the topmost part of the elasto-plastic layer. Finally, note that the amplitude of the stress invariant in the thickness evolution model is higher during the period of rising eccentricity, while smaller when the eccentricity is decreasing, since the sign of the non-hydrostatic pressure increases the yield stress during compressions, but decreases during extension.

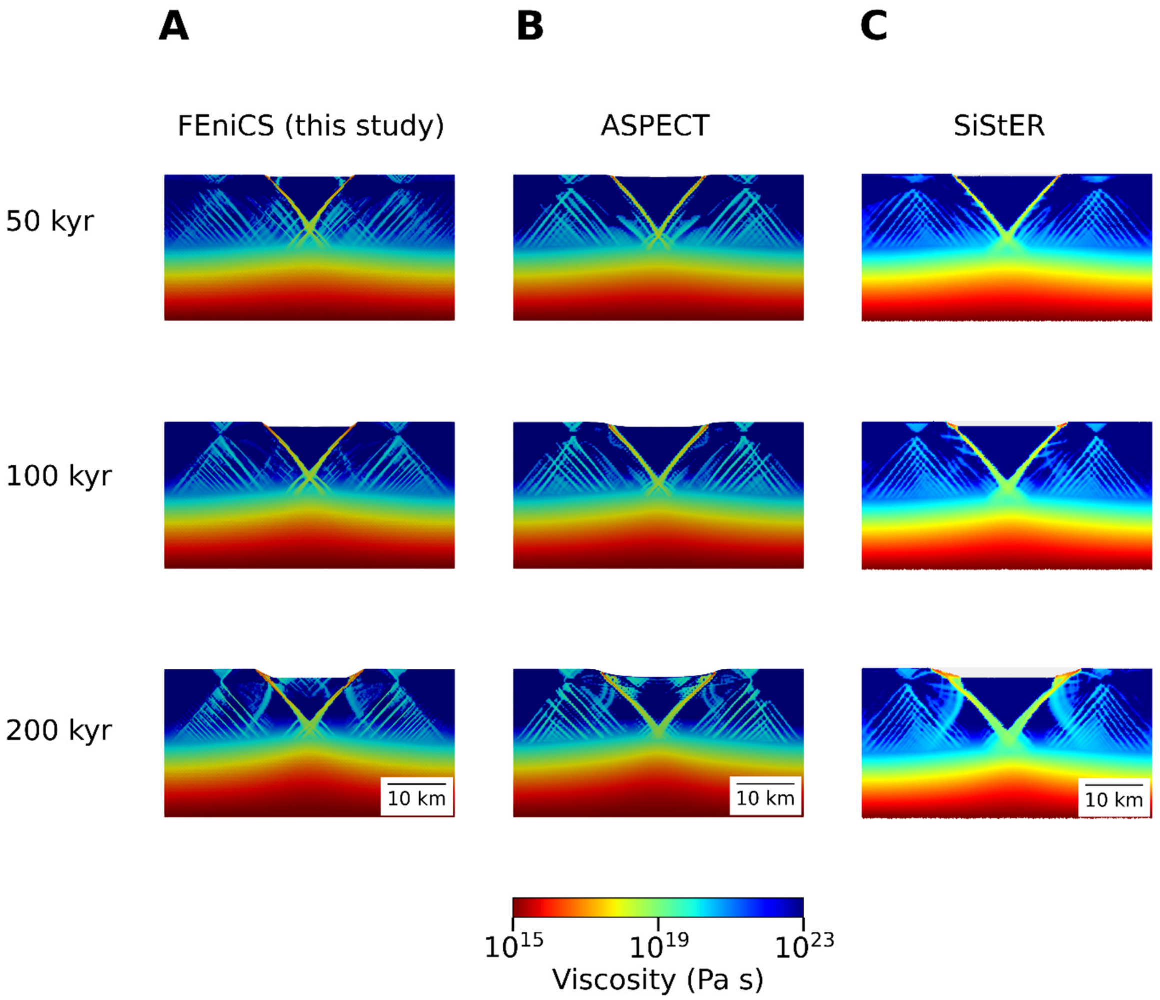

Fig. S1. Comparative simulation of ice shell extension. See Tables S1 and S3 for overview of parameters. **(A)** Solution by the numerical model presented in this study. **(B)** Solution by the ASPECT code. **(C)** Solution by the SiStER code.

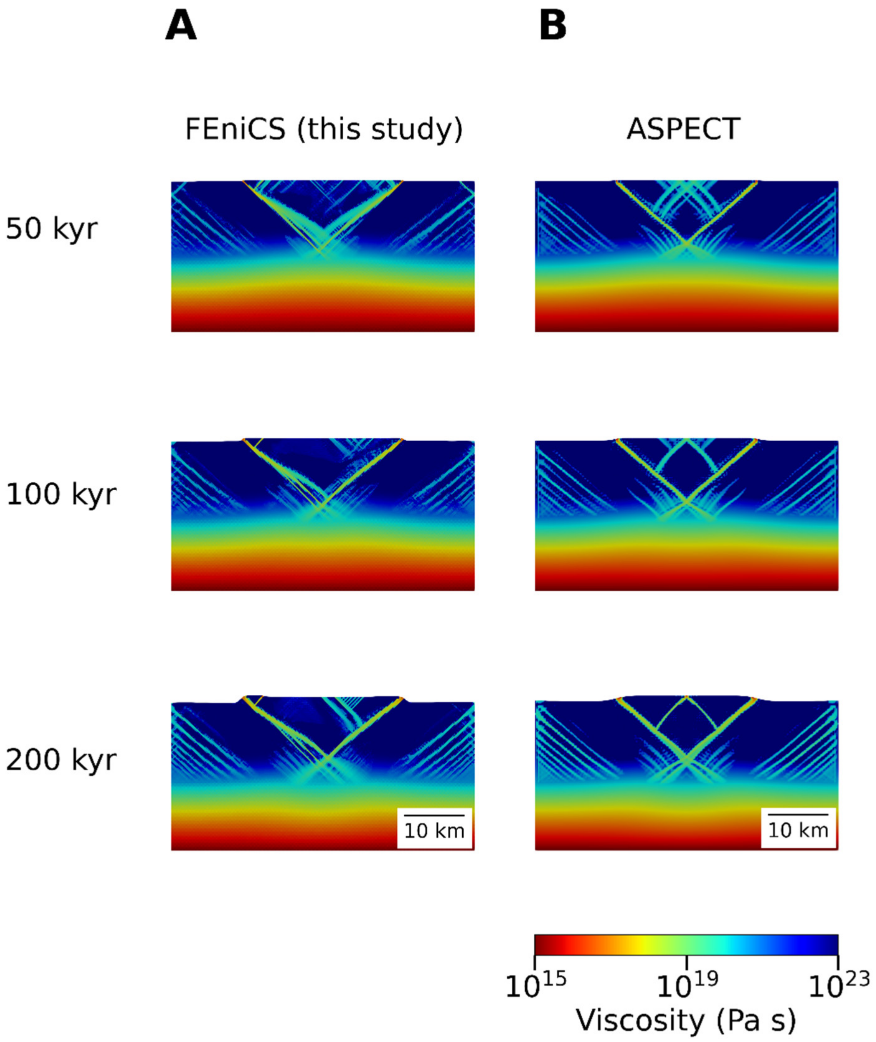

Fig. S2. Comparative simulation of ice shell compression. See Tables S1 and S3 for overview of parameters. **(A)** Solution by the numerical model presented in this study. **(B)** Solution by the ASPECT code. **(C)** Solution by the SiStER code.

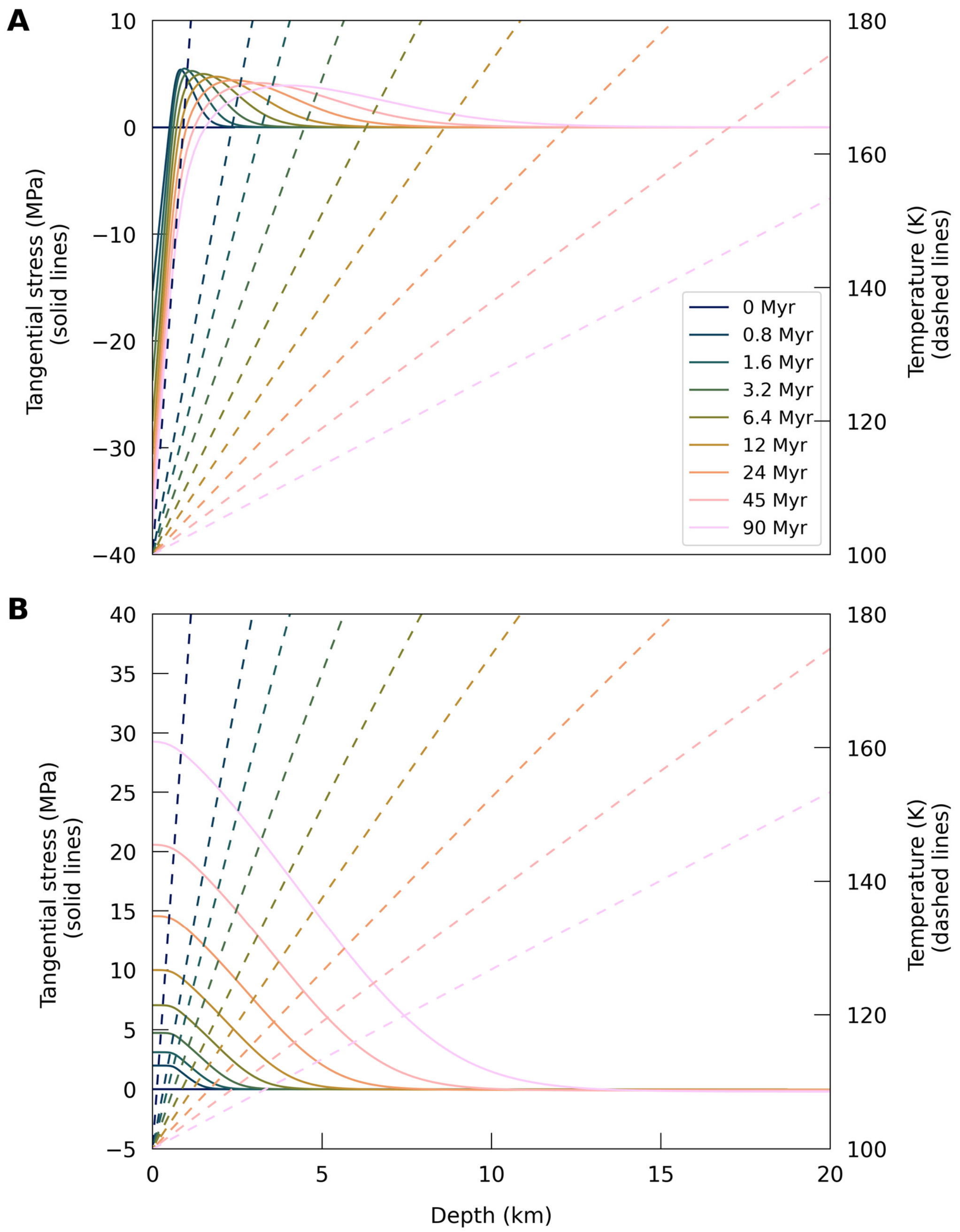

Fig. S3. Solution of stress evolution in a thickening ice shell. Following (52) and Eqs. 14 – 19 in Materials and Methods. **(A)** Stress due to cooling of the ice shell (temperature change effect). **(B)** Stress due to expansion of the hydrosphere (volume change effect). Compare with Figs. 1a and 2a in (25) and Figs. S5a and S6a in (14).

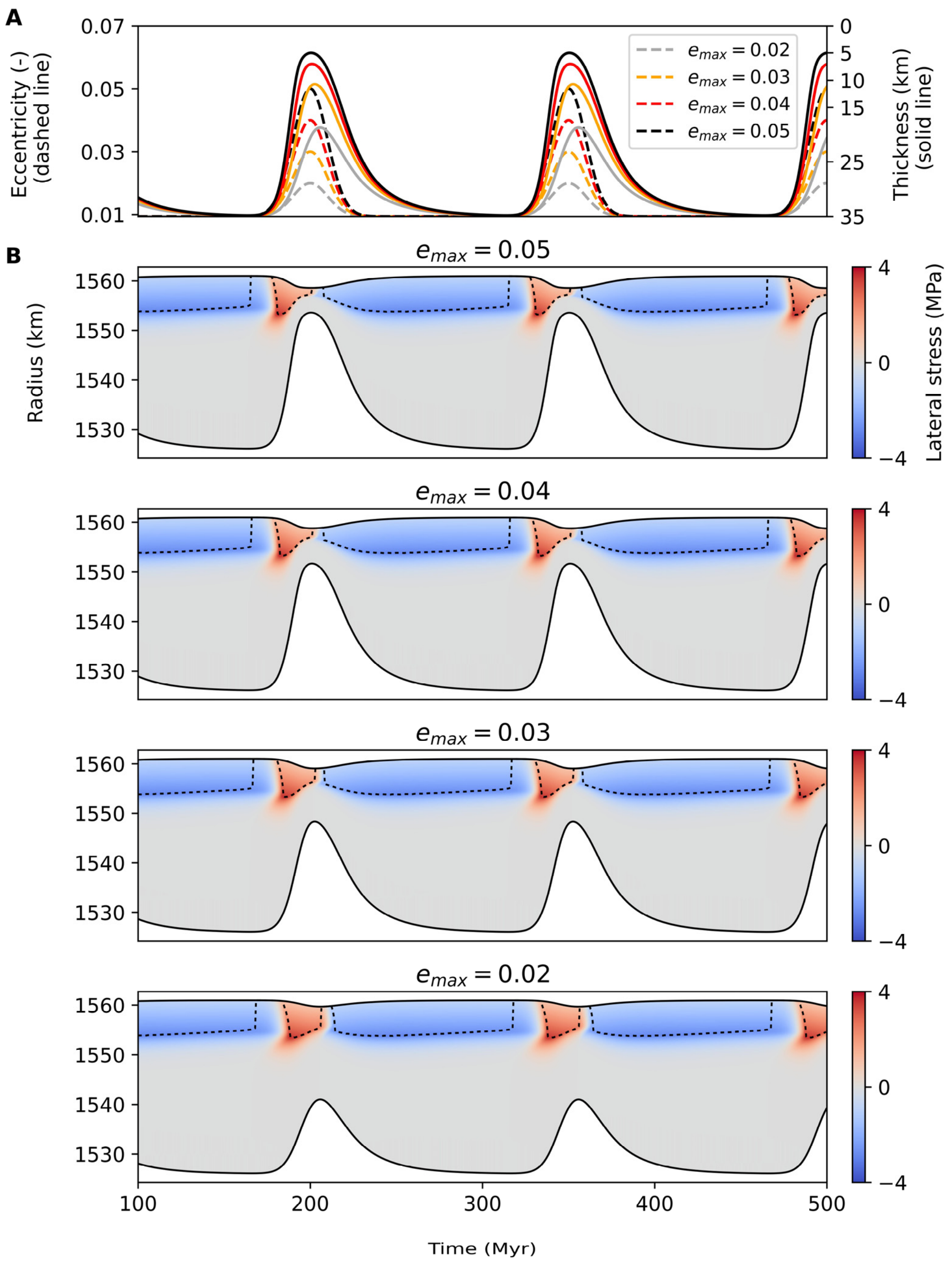

Fig. S4. **Time evolution of compressive stress in Europa's ice shell for different values of maximum eccentricity. (A)** Prescribed orbital eccentricity (left axis, dashed lines) and evolution of ice shell thickness (right axis, solid lines). **(B)** Time evolution of lateral component of the deviatoric stress ($\sigma_{\theta\theta}$ and equally $\sigma_{\varphi\varphi}$) in Europa's ice shell. Red and blue colors represent compressive and tensile stress, respectively. Dashed contour shows the deepest part of the shell where the yield criterion ($\sigma_{II} = \sigma_Y$) is met.

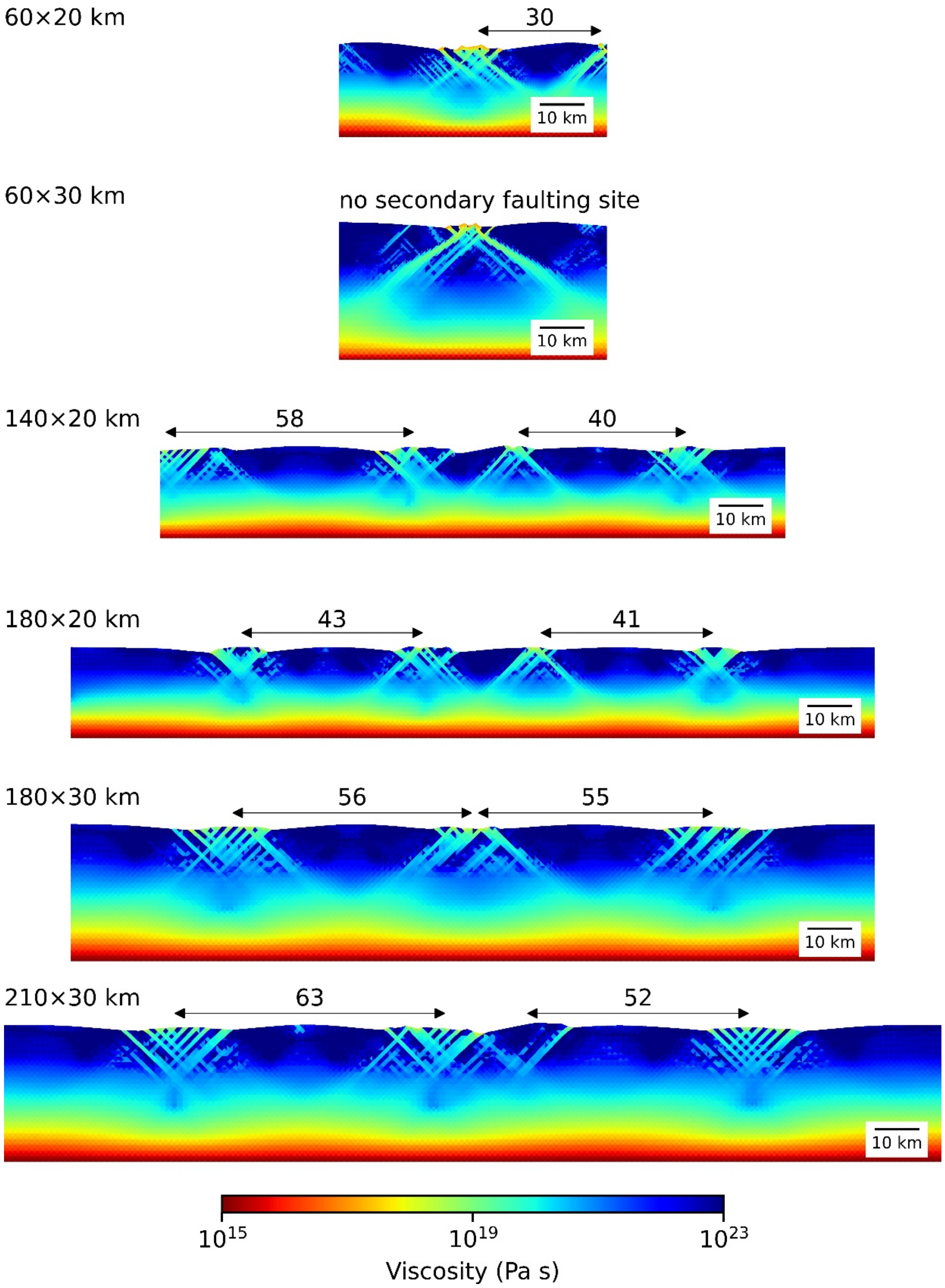

Fig. S5. Effect of the domain width on the emergence of secondary faulting sites. Arrows connect the centers of the primary and secondary faulting sites with the number above indicating their distance in kilometers.

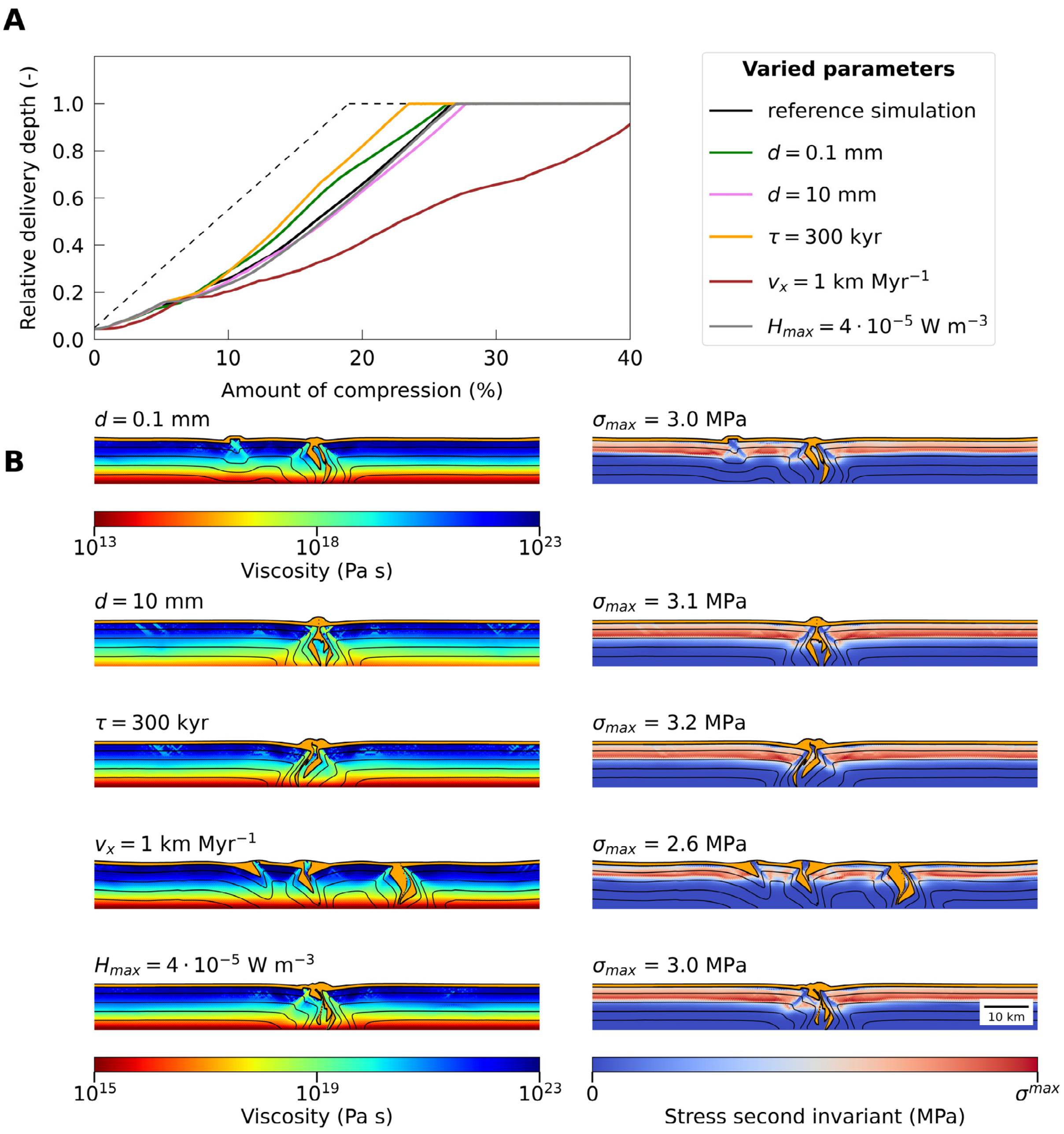

Fig. S6. Effect of secondary parameters on the evolution of relative delivery depth. Here, d is the grain size, τ is the healing time scale, v_x is the compression velocity and $H_{\max}$ is the maximum tidal dissipation rate. **(A)** Solid lines show the relative depth of the near-surface ice, dashed line shows the optimal efficiency (i.e. if the descent and compressional velocity were equal). **(B)** Snapshots of 10-km-thick ice shells at the moment of the first delivery of near-surface ice to the subsurface ocean.

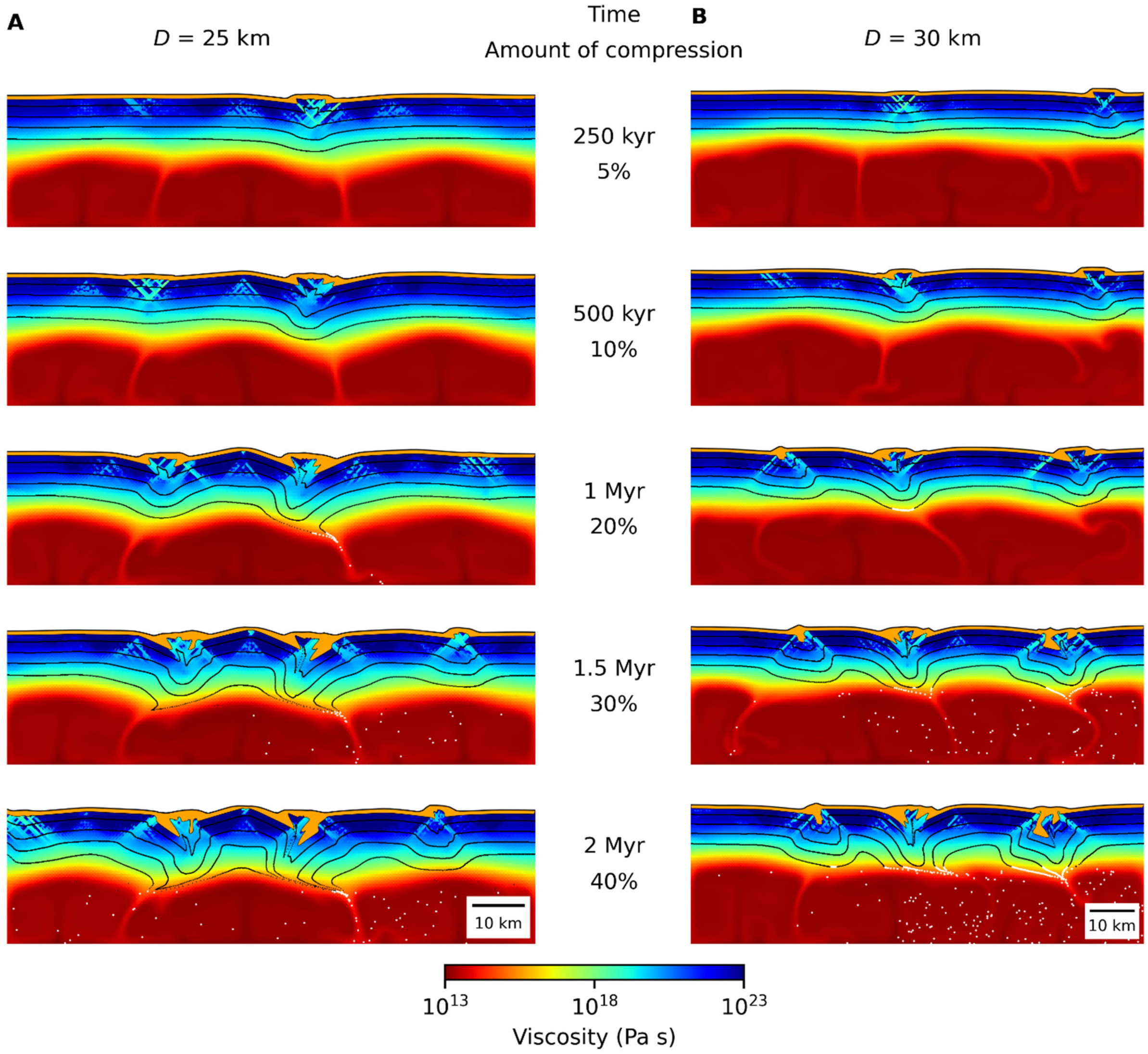

Fig. S7. **Time evolution of convective simulations. (A)** Shell thickness 25 km. **(B)** Shell thickness 30 km. As the ice that initially constituted the stagnant lid (black markers) proceeds towards the convective region, it gets captured into the convective flow (white markers).

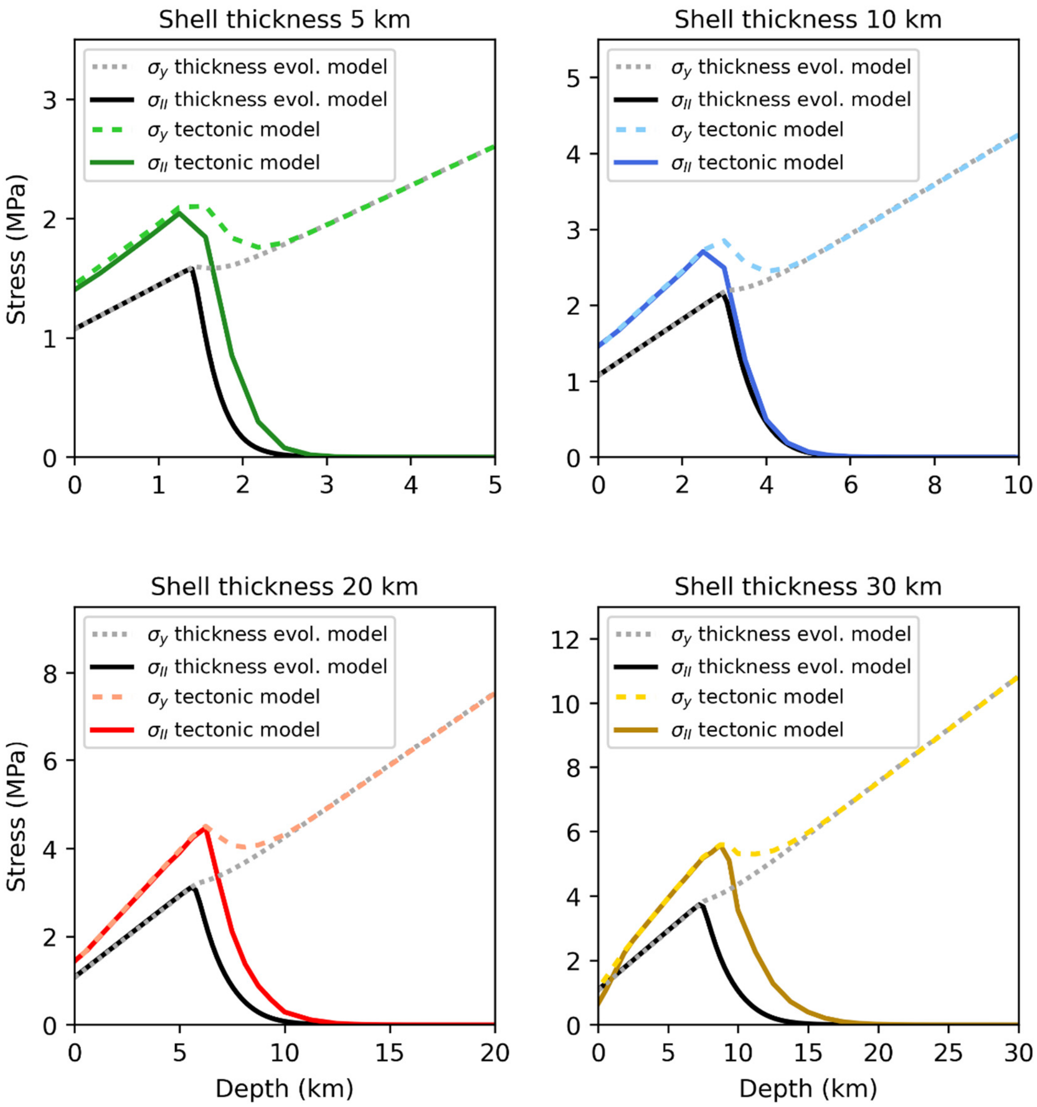

Fig. S8. **Comparison of stress profiles between the tectonic and the thickness evolution models.** Profiles from the thickness evolution model correspond to the moment when the shell had given thickness during the first compression phase. Profiles from the tectonic model correspond to the beginning of the simulation, when plastic deformation only started to occur.

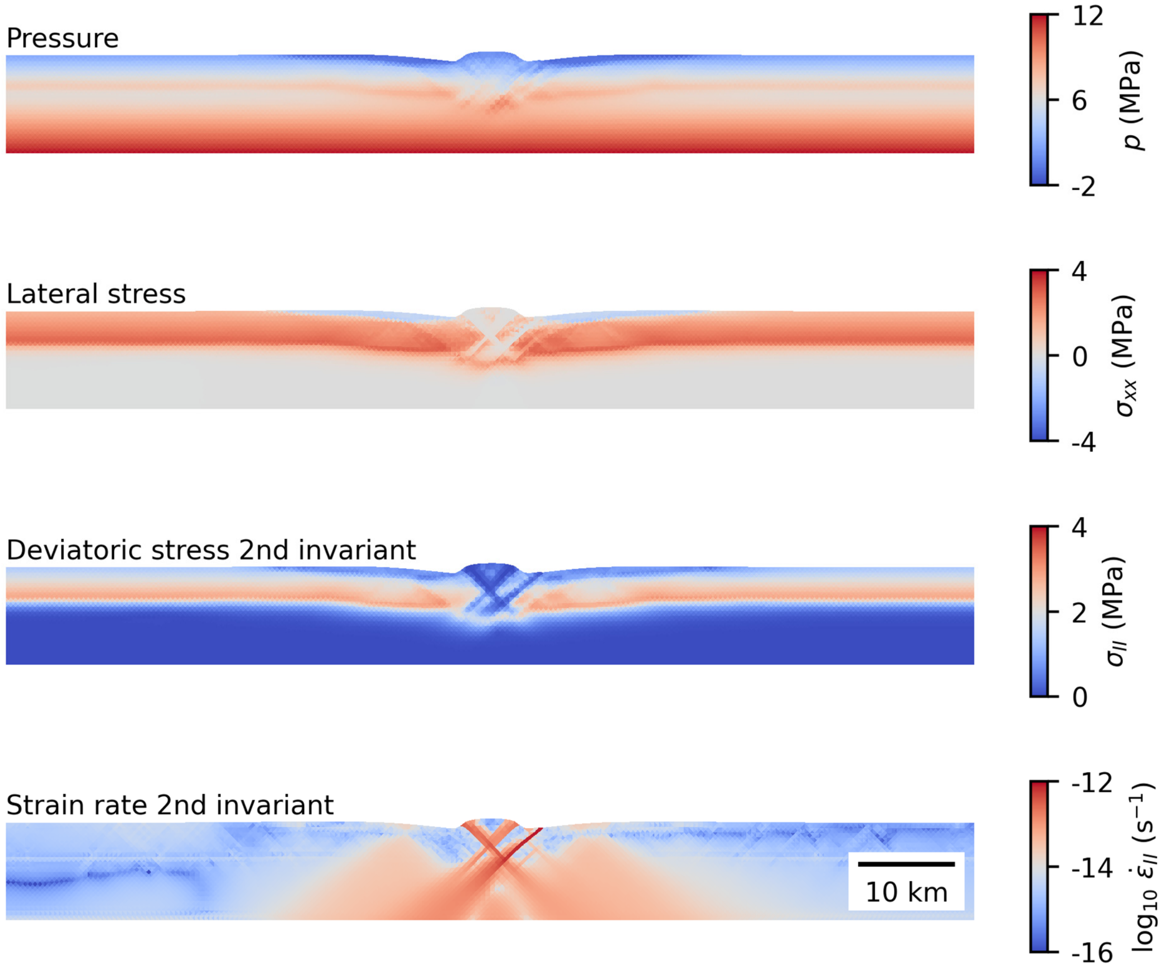

Fig. S9. **Additional properties from the tectonic model of lateral compression.** The snapshot corresponds to the last panel of Fig. 1A in the main text. Red and blue colors in the panel with lateral stress represent compressive and tensile stress, respectively.

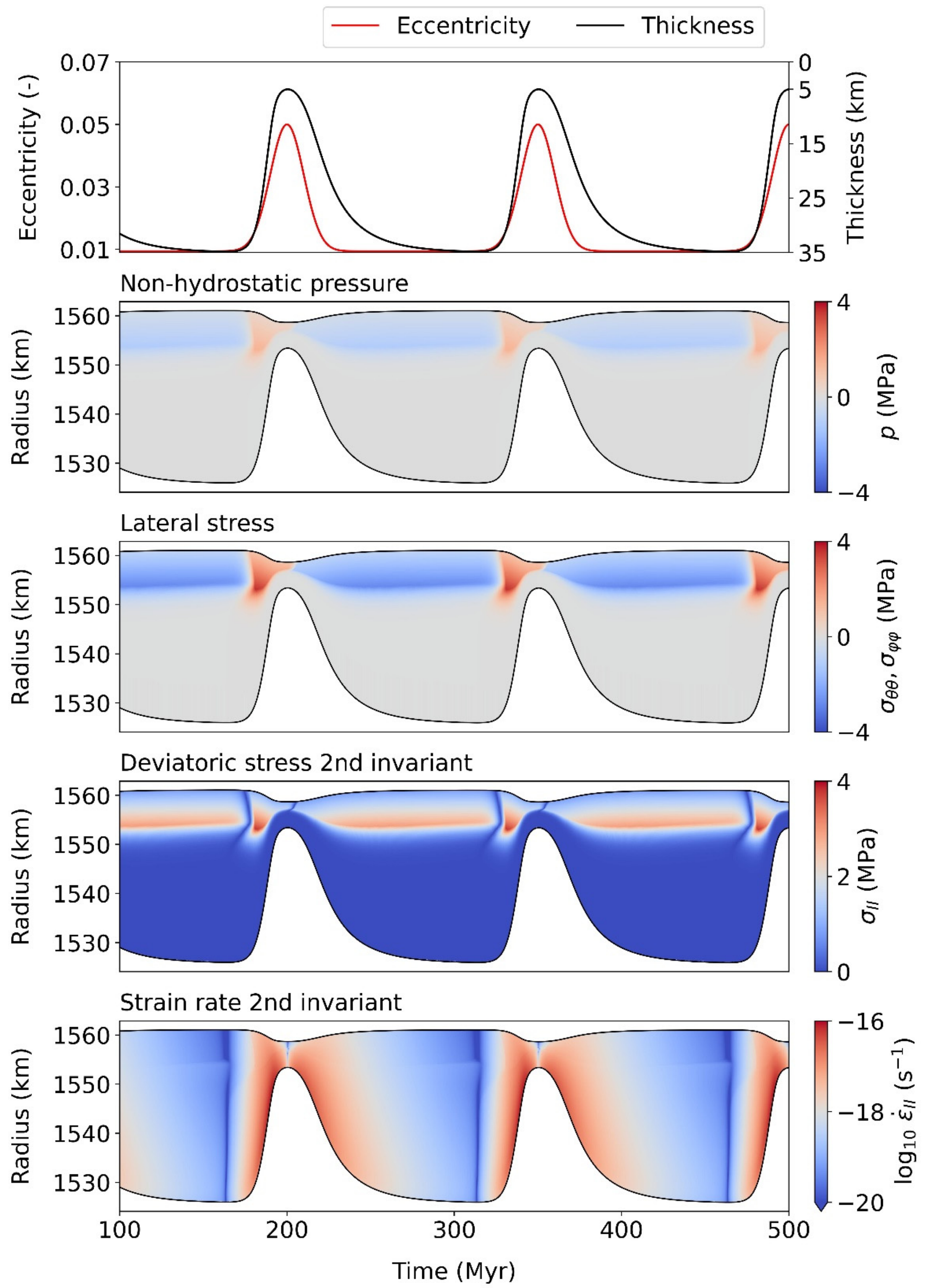

Fig. S10. **Additional properties from the thickness evolution model.** These properties correspond to the simulation presented in Fig. 5 in the main text.

Parameter	Description	Value	Unit
L	domain width	100	km
D	domain thickness	5 – 30	km
g	gravity	1.3	$\mathrm{m\ s^{-2}}$
T_{top}	temperature at surface	95	K
T_{bot}	temperature at ice-water interface	270	K
v_x	compression velocity	10	$\mathrm{mm\ yr^{-1}}$
**Rheological properties**			
d	grain size	1	mm
μ	shear modulus	3.52	GPa
C_0	cohesion of non-damaged ice	1	MPa
C_∞	cohesion of damaged ice	0	MPa
η_{cut}	cut-off viscosity	10^{23}	Pa s
ϕ	angle of internal friction	16	°
ε_∞	critical accumulated plastic strain	0.2	-
τ	cohesion healing timescale	∞	-
**Tidal heating properties**			
ω	Europa's orbital frequency	2×10^{-5}	$\mathrm{s^{-1}}$
H_{max}	tidal dissipation amplitude	4×10^{-6}	$\mathrm{W\ m^{-3}}$

Table S1. Overview of parameters for the tectonic model of lateral compression.

**Stress-independent**				
	D_0 (m^2 s^{-1})		Q (kJ mol^{-1})	
Volume diffusion	9.1×10^4		59.4	
Grain boundary diffusion	6.4×10^4		49	
**Stress-dependent**				
	A (Pa^{-n} m^p s^{-1})	n	p	Q (kJ mol^{-1})
Dislocation ($T < 258$ K)	4.0×10^{-19}	4	0	60
Dislocation ($T > 258$ K)	6.0×10^4	4	0	180
GBS ($T < 255$ K)	6.2×10^{-14}	1.8	1.4	49
GBS ($T > 255$ K)	5.6×10^{15}	1.8	1.4	192
BS	2.2×10^{-7}	2.4	0	60

Table S2. Parameters for viscous deformation mechanisms (19).

Parameter	Description	Value	Unit
L	domain width	50	km
D	domain thickness	25	km
Δt	time step	0.5	kyr
**Rheological properties**			
A	viscosity prefactor	2×10^{-13}	$\mathrm{Pa^{-1}\ s^{-1}}$
Q	activation energy	60	$\mathrm{kJ\ mol^{-1}}$
**Thermal properties**			
k	thermal conductivity	2.1	$\mathrm{W\ m^{-1}\ K^{-1}}$
c_p	specific heat	2100	$\mathrm{J\ kg^{-1}\ K^{-1}}$
α	thermal expansivity	1.6×10^{-4}	$\mathrm{K^{-1}}$

Table S3. Overview of additional/modified parameters for the comparative tectonic simulations presented in Figs. S1 and S2.

Parameter	Description	Value	Unit
r_s	initial surface radius	1561	km
r_b	initial ice-water interface radius	1526	km
ρ_w	water density	1000	$\mathrm{kg\ m^{-3}}$
L	latent heat	334	$\mathrm{kJ\ kg^{-1}}$
α_l	linear thermal expansivity coefficient	7.02×10^{-5}	$\mathrm{K^{-1}}$
P_{rad}	radiogenic heat production in the mantle	250	GW
P_{tidal}	tidal heat production in the mantle	106	GW
e_0	present-day eccentricity	0.0094	-
$e_{\max}$	maximum eccentricity	0.02 – 0.05	-
ν	Poisson’s ratio	0.3	-
μ	shear modulus	3.52	GPa

Table S4. Overview of parameters for the thickness evolution model. We consider value of the thermal expansivity coefficient at the mean temperature in the elastic lithosphere at 137.5 K (45, 46).

REFERENCES AND NOTES

1. R. J. Stern, SUBDUCTION ZONES. *Rev. Geophys.* **40**, 3-1–3-38 (2002).

2. R. Greenberg, The evil twin of Agenor: Tectonic convergence on Europa. *Icarus* **167**, 313–319 (2004).

3. L. Mével, E. Mercier, Resorption process in Astypalaea Linea extensive region (Europa). *Planet. Space Sci.* **53**, 771–779 (2005).

4. G. W. Patterson, J. W. Head, R. T. Pappalardo, Plate motion on Europa and nonrigid behavior of the icy lithosphere: The Castalia Macula region. *J. Struct. Geol.* **28**, 2237–2258 (2006).

5. S. A. Kattenhorn, L. M. Prockter, Evidence for subduction in the ice shell of Europa. *Nat. Geosci.* **7**, 762–767 (2014).

6. G. C. Collins, G. W. Patterson, C. E. Detelich, L. M. Prockter, S. A. Kattenhorn, C. M. Cooper, A. R. Rhoden, B. B. Cutler, S. R. Oldrid, R. P. Perkins, C. A. Rezza, Episodic plate tectonics on Europa: Evidence for widespread patches of mobile-lid behavior in the Antijovian memisphere. *J. Geophys. Res. Planets* **127**, e2022JE007492 (2022).

7. G. W. Ojakangas, D. J. Stevenson, Thermal state of an ice shell on Europa. *Icarus* **81**, 220–241 (1989).

8. G. Tobie, G. Choblet, C. Sotin, Tidally heated convection: Constraints on Europa's ice shell thickness. *J. Geophys. Res. Planets* **108**, (2003).

9. S. M. Howell, The likely thickness of Europa's icy shell. *Planet. Sci. J.* **2**, 129 (2021).

10. S. E. Billings, S. A. Kattenhorn, The great thickness debate: Ice shell thickness models for Europa and comparisons with estimates based on flexure at ridges. *Icarus* **177**, 397–412 (2005).

11. K. Sládková, O. Souček, K. Kalousová, M. Běhounková, Tidal walking on Europa's strike-clip faults—Insight from numerical modeling. *J. Geophys. Res. Planets* **125**, e2019JE006327 (2020).

12. H. Hussmann, T. Spohn, Thermal-orbital evolution of Io and Europa. *Icarus* **171**, 391–410 (2004).

13. S. M. Howell, R. T. Pappalardo, Band formation and ocean-surface interaction on Europa and Ganymede. *Geophys. Res. Lett.* **45**, 4701–4709 (2018).

14. M. L. Rudolph, M. Manga, M. Walker, A. R. Rhoden, Cooling crusts create concomitant cryovolcanic cracks. *Geophys. Res. Lett.* **49**, e2021GL094421 (2022).

15. S. Labrosse, A. Morison, R. Deguen, T. Alboussière, Rayleigh–Bénard convection in a creeping solid with melting and freezing at either or both its horizontal boundaries. *J. Fluid Mech.* **846**, 5–36 (2018).

16. M. Kihoulou, O. Čadek, J. Kvorka, K. Kalousová, G. Choblet, G. Tobie, Topographic response to ocean heat flux anomaly on the icy moons of Jupiter and Saturn. *Icarus* **391**, 115337 (2023).

17. J. Kvorka, O. Čadek, The role of subsurface ocean dynamics and phase transitions in forming the topography of icy moons. *Icarus* **412**, 115985 (2024).

18. M. M. Stempel, A. C. Barr, R. T. Pappalardo, Model constraints on the opening rates of bands on Europa. *Icarus* **177**, 297–304 (2005).

19. D. Goldsby, D. Kohlstedt, Superplastic deformation of ice: Experimental observations. *J. Geophys. Res. Solid Earth* **106**, 11017–11030 (2001).

20. A. N. B. Poliakov, W. R. Buck, "Mechanics of stretching elastic-plastic-viscous layers: Applications to slow-spreading mid-ocean ridges" in *Faulting and Magmatism at Mid-Ocean Ridges* [American Geophysical Union (AGU), 1998], pp. 305–323.

21. M. T. Bland, W. B. McKinnon, Forming Europa's folds: Strain requirements for the production of large-amplitude deformation. *Icarus* **221**, 694–709 (2012).

22. M. T. Bland, W. B. McKinnon, Does folding accommodate Europa's contractional strain? The effect of surface temperature on fold formation in ice lithospheres. *Geophys. Res. Lett.* **40**, 2534–2538 (2013).

23. R. T. Pappalardo, J. W. Head, R. Greeley, R. J. Sullivan, C. Pilcher, G. Schubert, W. B. Moore, M. H. Carr, J. M. Moore, M. J. S. Belton, D. L. Goldsby, Geological evidence for solid-state convection in Europa's ice shell. *Nature* **391**, 365–368 (1998).

24. B. C. Johnson, R. Y. Sheppard, A. C. Pascuzzo, E. A. Fisher, S. E. Wiggins, Porosity and salt content determine if subduction can occur in Europa's ice shell. *J. Geophys. Res. Planets* **122**, 2765–2778 (2017).

25. F. Nimmo, Stresses generated in cooling viscoelastic ice shells: Application to Europa. *J. Geophys. Res. Planets* **109**, (2004).

26. E. B. Bierhaus, K. Zahnle, C. R. Chapman, "Europa's crater distributions and surface ages" in *Europa*, R. T. Pappalardo, W. B. McKinnon, and K. K. Khurana, Eds. (Univ. Arizona Press, 2009), pp. 161–180.

27. T. Doggett, R. Greeley, P. Figueredo, K. Tanaka, "Geologic Stratigraphy and Evolution of Europa's Surface" in *Europa* (eds R. T. Pappalardo, W. B. McKinnon and K. K. Khurana) (Univ. Arizona Press, 2009), pp. 137–160.

28. P. H. Figueredo, R. Greeley, Resurfacing history of Europa from pole-to-pole geological mapping. *Icarus* **167**, 287–312 (2004).

29. S. M. Howell, R. T. Pappalardo, Can Earth-like plate tectonics occur in ocean world ice shells? *Icarus* **322**, 69–79 (2019).

30. A. R. Sarid, R. Greenberg, G. V. Hoppa, T. Hurford, B. R. Tufts, P. Geissler, Polar wander and surface convergence of Europa's ice shell: Evidence from a survey of strike-slip displacement. *Icarus* **158**, 24–41 (2002).

31. S. Kattenhorn, T. Hurford, "Tectonics of Europa" in *Europa*, R. T. Pappalardo, W. B. McKinnon, and K. K. Khurana, Eds. (Univ. Arizona Press, 2009), pp. 283–327.

32. T. Duretz, R. de Borst, P. Yamato, Modeling lithospheric deformation using a compressible visco-elasto-viscoplastic rheology and the effective viscosity approach. *Geochem. Geophys.* **22**, e2022GC010675 (2021).

33. K. Kalousová, O. Souček, G. Tobie, G. Choblet, O. Čadek, Water generation and transport below Europa's strike-slip faults. *J. Geophys. Res. Planets* **121**, 2444–2462 (2016).

34. J. R. Szalay, F. Allegrini, R. W. Ebert, F. Bagenal, S. J. Bolton, S. Fatemi, D. J. McComas, A. Pontoni, J. Saur, H. T. Smith, D. F. Strobel, S. D. Vance, A. Vorburger, R. J. Wilson, Oxygen production from dissociation of Europa's water-ice surface. *Nat. Astron.* **8**, 567–576 (2024).

35. K. P. Hand, R. W. Carlson, C. F. Chyba, Energy, chemical disequilibrium, and geological constraints on Europa. *Astrobiology* **7**, 1006–1022 (2007).

36. R. Greenberg, Transport rates of radiolytic substances into Europa's ocean: Implications for the potential origin and maintenance of life. *Astrobiology* **10**, 275–283 (2010).

37. S. D. Vance, K. P. Hand, R. T. Pappalardo, Geophysical controls of chemical disequilibria in Europa. *Geophys. Res. Lett.* **43**, 4871–4879 (2016).

38. M. Běhounková, G. Tobie, G. Choblet, M. Kervazo, M. Melwani Daswani, C. Dumoulin, S. D. Vance, Tidally induced magmatic pulses on the oceanic floor of Jupiter's moon Europa. *Geophys. Res. Lett.* **48**, e2020GL090077 (2021).

39. M. J. Russell, L. M. Barge, R. Bhartia, D. Bocanegra, P. J. Bracher, E. Branscomb, R. Kidd, S. McGlynn, D. H. Meier, W. Nitschke, T. Shibuya, S. Vance, L. White, I. Kanik, The drive to life on wet and icy worlds. *Astrobiology* **14**, 308–343 (2014).

40. A. Logg, K.-A. Mardal, G. Wells, Eds., *Automated Solution of Differential Equations by the Finite Element Method* (Springer-Verlag, 2012).

41. M. Alnæs, J. Blechta, J. Hake, A. Johansson, B. Kehlet, A. Logg, C. Richardson, J. Ring, M. E. Rognes, G. N. Wells, The FEniCS Project Version 1.5. *Archive of Numerical Software* **3**, 9–23 (2015).

42. W. B. Durham, L. A. Stern, Rheological properties of water ice-applications to satellites of the outer planets. *Annu. Rev. Earth Planet. Sci.* **29**, 295–330 (2001).

43. L. L. Lavier, W. R. Buck, A. N. B. Poliakov, Factors controlling normal fault offset in an ideal brittle layer. *J. Geophys. Res. Solid Earth* **105**, 23431–23442 (2000).

44. V. Patočka, H. Čížková, P. Tackley, Do elasticity and a free surface affect lithospheric stresses caused by upper-mantle convection? *Geophys. J. Int.* **216**, 1740–1760 (2019).

45. K. Röttger, A. Endriss, J. Ihringer, S. Doyle, W. F. Kuhs, Lattice constants and thermal expansion of H2O and D2O ice Ih between 10 and 265 K. *Acta Crystall. B* **50**, 644–648 (1994).

46. R. Feistel, W. Wagner, A new equation of state for H_2O ice Ih. *J. Phys. Chem. Ref. Data.* **35**, 1021–1047 (2006).

47. P. V Hobbs, *Ice Physics* (OUP, 2010).

48. T. B. McCord, C. Sotin, Ceres: Evolution and current state. *J. Geophys. Res. Planets* **110**, E05009 (2005).

49. R. Deguen, T. Alboussière, P. Cardin, Thermal convection in Earth's inner core with phase change at its boundary. *Geophys. J. Int.* **194**, 1310–1334 (2013).

50. T. Alboussière, R. Deguen, M. Melzani, Melting-induced stratification above the Earth's inner core due to convective translation. *Nature* **466**, 744–747 (2010).

51. J. B. Holt, Thermal diffusivity of olivine. *Earth Planet. Sci. Lett.* **27**, 404–408 (1975).

52. D. A. Varshalovich, A. N. Moskalev, V. K. Khersonskii, *Quantum Theory of Angular Momentum* (World Scientific, 1988).

53. B. Aygün, O. Čadek, Impact of the core deformation on the tidal heating and flow in Enceladus' subsurface ocean. *J. Geophys. Res. Planets* **128**, e2023JE007907 (2023).

54. J. Donea, A. Huerta, J.-P. Ponthot, A. Rodriguez-Ferran: *Arbitrary Lagrangian-Eulerian Methods* (Wiley, 2004).

55. M. Kronbichler, T. Heister, W. Bangerth, High accuracy mantle convection simulation through modern numerical methods. *Geophys. J. Int.* **191**, 12–29 (2012).

56. T. Heister, J. Dannberg, R. Gassmöller, W. Bangerth, High accuracy mantle convection simulation through modern numerical methods – II: Realistic models and problems. *Geophys. J. Int.* **210**, 833–851 (2017).

57. W. Bangerth, J. Dannberg, M. Fraters, R. Gassmoeller, A. Glerum, T. Heister, R. Myhill, J. Naliboff, Aspect v2.4.0, *Zenodo* (2022).

58. J.-A. Olive, M. D. Behn, E. Mittelstaedt, G. Ito, B. Z. Klein, The role of elasticity in simulating long-term tectonic extension. *Geophys. J. Int.* **205**, 728–743 (2016).
